# Maize leaf endosphere microbiome was affected by domestication and shows patterns consistent with microbial dysbiosis

**DOI:** 10.3389/frmbi.2026.1735358

**Published:** 2026-02-23

**Authors:** Ilksen Topcu, Julio S. Bernal, Sanjay Antony-Babu

**Affiliations:** 1 Department of Plant Pathology and Microbiology, Texas A&M University, College Station, TX, United States; 2 Department of Entomology, Texas A&M University, College Station, TX, United States

**Keywords:** leaf endosphere, maize, microbiome, plant domestication, teosinte, *Zea diploperennis*, *Zea mays mays*, *Zea mays parviglumis*

## Abstract

**Background:**

Whether domestication, post-domestication spread, and breeding affected the maize leaf endosphere microbiota is poorly understood despite the well-known effects of those processes on the crop’s genetics and responses to the environment. We examined the leaf endosphere microbial communities associated with three plant groups (Zea mays): teosintes, maize landraces, and maize elite inbreds. The teosintes group included Balsas (Z. mays parviglumis) and perennial (Zea diploperennis) teosinte, and each maize (Z. mays mays) group included genotypes from Mexico and USA. We used 16S-V4 region amplicon sequencing of the leaf endophytic microbiota to infer how the microbial communities of inbred maize may have been shaped by the crop’s evolution, and whether they were affected by: (i) the transition from a perennial life history to an annual life history in the teosintes; (ii) domestication of maize from Balsas teosinte; (iii) northward spread of landrace maize from Mexico to the US; and (iii) breeding of landrace maizes to produce elite inbreds.

**Results:**

The leaf endophytic microbial community differed among the plant groups and genotypes, and was affected by domestication, as indicated by a decline in bacterial diversity and changes in microbial community structure between wild (teosinte) and domesticated (maize) Zea. While the microbial community structure was stringent and regulated in the teosintes, it was variable in the maize landraces and inbreds, as evidenced by greater distances to centroid based on Euclidean dissimilarity metric. This pattern was suggestive of microbial dysbiosis in the leaf endosphere associated with domestication and is consistent with predictions of the Anna Karenina principle. This finding marks the first evidence of dysbiosis associated with domestication. FAPROTAX predictions suggested that the teosintes may harbor microbial communities enriched in taxa associated with cellulolytic, chitinolytic, and nitrate respiration functions, while the maizes showed higher fermentation and nitrate reduction functions.

**Conclusions:**

Our results showed that the leaf endosphere microbial community structures in maize are consistent with alterations associated with dysbiosis. Our findings enhanced our understanding of the effects of anthropogenic processes including crop domestication, spread, and breeding on the leaf endosphere of elite maize cultivars, and may guide the development of evolutionarily-and ecologically sustainable biofertilizers and biocontrol agents.

## Background

The plant microbiome consists of numerous taxa of microorganisms, including bacteria, fungi, archaea, protists, and viruses, which play essential roles in many plant functions, such as growth, nutrient uptake, plant resistance against pathogens and insects, and plant tolerance of abiotic stresses (Vandenkoornhuyse et al., 2002; Hassani et al., 2018; Trivedi et al., 2020; Sahu et al., 2022). The microbial components of plant holobiomes coevolved with their host plants and occurs in specialized niches, such as the rhizosphere (space nearest plant roots that is inhabited by microorganisms), phyllosphere (surface of aboveground plant tissues that is inhabited by microorganisms), and endosphere (internal above- and belowground plant tissues) (Hassani et al., 2018). The structure and composition of microbiome communities colonizing plant niches can be influenced by numerous variables (e.g., plant genotype, soil type, biotic and abiotic environmental variables, plant development stage, and geographical location), and plant survival and reproduction are mediated by microbiome communities (Finkel et al., 2011; Jackson and Denney, 2011; Johnston-Monje et al., 2014; Brisson et al., 2019; Xiong et al., 2021a). Moreover, host plants selectively recruit particular beneficial microorganisms in their niche compartments and, in return, the recruited microbial assemblages enhance the survival and reproduction of their hosts (Fitzpatrick et al., 2019; Xiong et al., 2021b). For example, rhizosphere microbiota is known to play essential roles in soil nutrient acquisition and enhance plant defense against biotic stressors (Bulgarelli et al., 2013; Begum et al., 2019; Fitzpatrick et al., 2019). Similarly, phyllosphere and leaf endosphere microbiota play crucial roles in defense against plant pathogens and other important plant processes (Ryan et al., 2008; Balint-Kurti et al., 2010; Johnston-Monje and Raizada, 2011; Shehata et al., 2016; Sahu et al., 2022).

The processes of plant domestication, geographic spread, and improvement resulted in significant reductions in the genetic diversity of crop species and shaped their microbial assemblages (Szoboszlay et al., 2015; Pérez-Jaramillo et al., 2016; Hassani et al., 2020; Gutierrez and Grillo, 2022; Huang et al., 2022). Maize (*Zea mays mays*) is one of the most widely cultivated cereal crops globally and the Americas is the crop’s top-producing region (FAO, 2022). Maize is the product of Balsas teosinte (*Zea mays parviglumis*) domestication, which began ca. 9,200 years ago in the Pacific lowlands of southern Mexico (Matsuoka et al., 2002). Several post-domestication processes, including farmer (artificial) and natural selection, geographic spread, and modern breeding led to dramatic morphological- and physiological trait changes in maize (Yamasaki et al., 2005; Zhang et al., 2023). Artificial selection by farmers after domestication produced a wide variety of landrace cultivars (Wellhausen, 1952; Vielle-Calzada and Padilla, 2009). Dispersal to North America and the present-day USA began as early as 2,100 years ago and was followed by *in situ* development of landrace cultivars, followed by synthetic cultivars derived from simple crossed of landraces in the 1800s, and modern elite inbred and hybrid cultivars beginning ca. 100 years ago (Vigouroux et al., 2008; Merrill et al., 2009; Van Heerwaarden et al., 2011; Hufford et al., 2012; van Heerwaarden et al., 2012). Genetic (allelic) diversity in maize has been reduced due to a population bottleneck and directional selection targeting genomic regions associated with agronomically significant traits (Zhang et al., 2023). Two prominent examples are tgal1 and ZmSRO1d-R. The tga1 largely controls the loss of the seed fruitcase. The fruitcase a critical and early step in maize domestication (Wang et al., 2005), where in Balsas teosinte, each seed is enclosed in a hardened fruitcase largely precluding human consumption of the seed. Domestication of Balsas teosinte required selection, and fixation, upon a single nonsynonymous G to C mutation in tga1 leading to the evolution of the fully exposed seeds of maize (Wang et al., 2015). The ZmSRO1d-R, which modulates the balance between crop yield and drought resistance by increasing ROS levels in guard cells, was selected during domestication and breeding (Gao et al., 2022). Previous study has employed genome-wide association studies (GWAS) to systematically examine how genetic variation in maize correlates with the composition of root-associated microbial communities. By analyzing large populations of maize together with their corresponding microbial profiles, researchers identified single-nucleotide polymorphisms (SNPs) associated with shifts in rhizosphere community structures (Meier et al., 2022). Although no study has yet explored how genetic loci in teosinte influence its microbiome composition, maize domestication has led to a significant reduction in genetic diversity relative to teosinte. This observation raises the question of whether this reduction in genetic diversity may, in turn, have impacted microbial diversity in the leaf endosphere.

Microbial taxa relevant to plant survival and reproduction may be absent or underrepresented in the microbial assemblages of modern crop cultivars, plausibly because such cultivars have been bred to thrive under conditions in which crop nutrition and defense against insects and pathogens depend on use of synthetic fertilizers and pesticides (Chaudhary, 2013; Gopal and Gupta, 2016; Pérez-Jaramillo et al., 2016). Hence it is important to evaluate whether and to which extent the microbial community structure in modern crops differs from the structures in their domesticated and wild ancestors, which typically thrive in the absence of synthetic fertilizers and pesticides. Particularly, it is relevant to evaluate whether crop microbial community structure was affected by domestication and post-domestication processes, e.g., farmer selection, geographic spread, and modern breeding, or whether signs of diminished microbial recruitment are evident. Additionally, it is important to evaluate whether microbial dysfunction, i.e., dysbiosis, is associated with domestication and post-domestication processes. Dysbiosis is defined as encompassing any microbial compositional or functional alteration, including changes in abundance, diversity, or critical functions​ (Bäckhed et al., 2012; Henao-Mejia et al., 2012; Olesen and Alm, 2016; Arnault et al., 2023). Healthy microbiomes are typically characterized by a balanced composition with controlled variability, where deterministic processes driven by host regulation maintain functional stability and resilience (Bäckhed et al., 2012; Lozupone et al., 2012; Henry et al., 2021). A reduction in heterogeneity may indicate dominance by a few microbial taxa, potentially resulting from environmental stressors or pathogen invasion, leading to functional impairments such as disease resistance (Bäckhed et al., 2012; Chen et al., 2020; Han et al., 2022). In addition, a rise in heterogeneity often represents increased stochasticity in microbial community assembly, associated with dysbiosis and reduced host capacity to regulate its microbiota​ (Bäckhed et al., 2012; Arnault et al., 2023). These shifts highlight the intricate relationship between microbiome structure and host health, where deviations from balanced heterogeneity can significantly impact fitness and productivity. Subjected to abiotic or biotic stresses, plant (and animal) microbiomes may fall under dysbiosis (Lavrinienko et al., 2020; Han et al., 2022; Arnault et al., 2023). The Anna Karenina Principle (AKP), viz. “all healthy microbiota are alike; each disease-associated microbiota is sick in its own way” (Zaneveld et al., 2017), is used in microbiome studies to describe whether plants harbor a dysfunctional microbiota (Zaneveld et al., 2017) (Ma, 2020),. While dysbiosis has been demonstrated well in human and animal microbiomes (Zaneveld et al., 2017; Ma, 2020), little is known in plant-associated microbiomes. Dysbiosis is linked to poor health in hosts (Sohrabi et al., 2023) and the current knowledge of plant microbiome dysbiosis, although limited, comes from the study of plant diseases (Koskella et al., 2017; Choi et al., 2020; Lee et al., 2021; Han et al., 2022). For instance, a study conducted on the rhizosphere of dead, but standing, Korean fir trees revealed a state of dysbiosis characterized by a noticeable decrease in the richness and diversity of the microbiota within the rhizosphere when compared to the microbiota of healthy trees (Han et al., 2022). Additionally, a defect in pattern-triggered immunity and the absence of a specific metabolite, previously linked to phyllosphere, are associated with dysbiosis in plants (Chen et al., 2020; Su et al., 2024). Understanding the underlying conditions that lead to microbial dysfunction, and fitness costs in plant hosts, is important because it may inform the study of beneficial microbiomes and the development of microbial inoculants for sustainable crop production.

The study presented here is the first, to the best of our knowledge, to assess whether and how domestication, geographic spread, and modern breeding affected the bacterial community of the maize leaf endosphere. Some effects of domestication and breeding on the microbial assembly of the maize rhizosphere were reported in prior studies (Favela et al., 2021; Huang et al., 2022). Here, we report on the assemblages of endophytic bacterial microbiota of leaves in a suite of teosintes (*Zea* spp. other than maize) and maize genotypes spanning the evolution of maize: perennial teosinte (*Zea diploperennis*), Balsas teosinte, landrace maize cultivars, and elite inbred maize cultivars. Using this suite, we inferred on effects of the perennial to annual life history transition in teosintes (perennial vs Balsas teosinte), domestication (Balsas teosinte vs. Mexican landrace maize), geographic spread (Mexican vs USA landrace maize), and modern breeding (Mexican and US landrace vs elite inbred maize). We addressed whether the structural and compositional aspects of the leaf endophytic bacterial community were mediated by life history evolution, domestication, geographic spread, and modern breeding, and whether dysbiosis is associated with domestication or other processes. Furthermore, we examine how stringency in teosinte and maize, defined as the level of selectivity or ecological filtering exerted by the plant host, has changed during the process of maize domestication. Numerous studies have documented marked losses of genetic and trait diversity associated with maize domestication (Buckler and Stevens, 2006; Ross-Ibarra et al., 2007; Hufford et al., 2012; Fontes-Puebla et al., 2021; Chen et al., 2022; Bernal et al., 2023) and we suggest that it is plausible that such losses parallel changes in the leaf endophytic bacterial community. We hypothesized that transition from perennial to annual life history in teosintes, and maize domestication, geographic spread, and breeding would each be associated with: i) reduction in the diversity and richness of the microbial community recruited within the leaf endosphere; and ii) reductions in stringency and increases in variability in processes mediating recruitment. We also investigated whether these changes were associated with signatures of dysbiosis in terms of alpha- and beta-diversities. This includes a reduction in alpha-diversity, such as decreased richness or diversity, and increased beta-diversity, reflecting greater stochasticity in community composition, consistent with the Anna Karenina Principle.

## Materials and methods

### Plant materials and growth

A suite of 17 teosinte and maize accessions corresponding to six genotypes contained within three plant groups were selected to represent the evolution of maize from perennial teosinte to elite inbred maize. Specifically, this suite included the following three plant groups, each with two genotypes: (i) teosinte, including perennial teosinte (1 accession) and Balsas teosinte (6 accessions); (ii) Mexican maize, including Mexican landrace maize (1 accession) and Mexican elite inbred maize (2 accessions); and (iii) US maize, including US landrace maize (3 accessions) and US elite inbred maize (4 accessions) (**Supplementary Table S1**). Using these plant groups and genotypes we addressed whether the leaf endosphere bacterial community was affected by: (i) the transition from a perennial to an annual life history (comparison: perennial vs Balsas teosinte); (ii) domestication (Balsas teosinte vs Mexican landrace); (iii) northward spread (Mexican landrace vs US landrace); (iv) breeding in Mexico (Mexican landrace vs Mexican elite inbred); and (v) breeding in USA (US landrace vs US elite inbred). Domestication effects were inferred using the maize landrace Tuxpenño because its distribution overlaps that of Balsas teosinte and with the area where maize was domesticated (Matsuoka et al., 2002; Yang et al., 2019).

Seeds were surface sterilized by soaking in 1% Tween20 followed by 5% bleach solution, both steps for one minute each. This was followed by alcohol wash with 70% ethanol twice in less than one minute and a final rinse with sterile reverse osmosis water (RO water) for one minute. The surface-sterilized seeds were transferred to sterile paper prewet with water in sterile Petri dishes for three days in a dark space for pre-germination. One germinated seed was transplanted per cone-tainer pot (4 × 25 cm, diam × length) that contained equal mixture (v/v) of play sand (Quikrete, Atlanta, Georgia, USA) and SunGro Sunshine LC1 soil mix (SunGro, Agawam, Massachusetts, USA). Before use, the soil-sand mixture was autoclaved thrice at 121 °C for 1 hour at 24hrs intervals to reduce the initial microbial load and recolonizing microbial community in the soil (King et al., 2024). Five replicates per accession (except CML277 with three replicates because of poor germination) were grown for 4 weeks in a growth room under artificial LED lights and 1-week greenhouse condition with natural light. The plants were watered with tap water every other day.

### Collection of leaf endosphere samples

Above-ground biomass was harvested from the 5-week-old seedlings. The entire above-ground mass was mostly made of leaf matter or curled up leaf with little or no real stem. The leaf samples were surface sterilized by soaking in a 5% bleach solution for one minute, a single rinse of 70% ethanol, and rinsed with sterile RO water. Surface-sterilized leaf samples were dabbed on sterile paper to remove excess moisture and sliced into small pieces. The sample tissues were flash-frozen and stored at -80 °C until further processing.

### DNA extraction

DNA was extracted from the leaf tissues using the ZymoBIOMICS™ DNA Miniprep Kit (Zymo Research, Irvine, CA, USA) and the protocol was modified (See detailed Method S1). DNA concentration was quantified using SpectraMax QuickDrop Micro-Volume Spectrophotometer, and the extracted DNA was verified by 1% agarose gel electrophoresis. According to the results of QuickDrop, the samples, which have less than 0.800 A260/A230 ratios, were tested whether they can be amplified by PCR (and hence can be used to produce metabarcoding libraries) with universal primers 27F (5′-AGAGTTTGATCATGGCTCAG-3′) as forward and 1492R (5′-GGTTACCTTGTTACGACTT-3′) as reverse primer (Lane, 1991). 10 μl PCR reaction was containing 5 μl of mix KAPA2G Fast HotStart ReadyMix PCR Kit (KAPA Biosystems, Wodurn, MA), 3 μl Molecular Biology Grade Water, CorningTM, 0.5 μl of each primer, and 1 μl of a 1e-2 DNA dilution from the leaf sample. PCR reaction mixture was amplified as follows: 95 °C for 3 mins, then 95 °C for 15 sec, 48 °C for 15 sec, and followed by 72 °C for 30 sec for 39 cycles. Subsequently, a final extension step was performed at 72 °C for 1 min, and the reaction was held at 10 °C continually. PCR products were run on 1% agarose gel and confirmed for the presence of bacterial 16S rRNA genes. DNA concentration was quantified using Qubit dsDNA HS Assay kit by Qubit 2.0 fluorometer (Life Technologies, Carlsbad, USA).

### Metabarcoding and Illumina Mi Seq sequencing

Metabarcoding library preparation and sequencing was carried out by TxGen - Genomics and Bioinformatics Services, Texas A&M University, College Station (https://www.txgen.tamu.edu). Briefly, the V4 region of the 16S rRNA gene was amplified using NEXTflex-16S V4 Amplicon-Seq Library Prep Kit 2.0 and primers 16S V4 forward (5’-GACGC TCTTC CGATC TTATG GTAAT TGTGT GCCAG CMGCC GCGGT AA-3’) and 16S V4 reverse (5’-TGTGC TCTTC CGATC TAGTC AGTCA GCCGG ACTAC HVGGG TWTCT AAT-3’) (BIOO Scientific, Austin, TX, USA). The libraries were sequenced using the Illumina MiSeq MCS 2.5.0.5 and RTA 1.18.54 software with default parameter settings. Sequencer.bcl basecall files were formatted into fastq files using bcl2fastq 2.20 script configureBclToFastq.pl. The quality of Illumina Mi Seq sequencing reads was assessed with FastQC (Andrews, 2010). Although no-template controls were not sequenced, negative controls were included during DNA extraction and PCR library preparation to ensure that the amplicons originated from the samples and not from the reagents. Additionally, microbial cultivation tests (data not shown) demonstrated that the surface-sterilized leaf samples contained sufficient microbial biomass for downstream analysis.

Raw amplicon data were processed using MOTHUR software (v.1.48.0, https://mothur.org/wiki/miseq_sop/, accessed online December 2022) following the default settings (Kozich et al., 2013). The recommended SOP was followed except for the maximum length adjusted to 320 to accommodate our assembled read lengths. Because this study was designed to investigate the bacterial community composition of the leaf endosphere, we performed “remove.lineage” command in mothur to filter out sequences associated with chloroplast, mitochondria, archaea, eukaryota, and unknown lineages. Sequences were clustered into operational taxonomic units (OTUs) at a 97% similarity threshold. Because this study focused on broad patterns of microbial community structure, diversity, and ecological variation across teosinte and maize, 97% OTU clustering was appropriate for these objectives. The consensus taxonomy of the OTUs was generated using the “classify.otu” command in mothur with reference data using the SILVA database (release 138.1, https://mothur.org/wiki/silva_reference_files/) (Pruesse et al., 2007).

### Statistical analyses

OTU data and taxonomic information on OTUs were analyzed for all statistical calculations and data visualization in R (R Core Team, 2022) and JMP Pro 17 statistical software (JMP®, 2025). The data were normalized using cumulative sum scaling (CSS) for all analyses, except for Venn diagram analysis and richness metrics, which were calculated based on absolute species counts. Richness metrics were calculated from OTU tables filtered to retain taxa with ≥10 total counts across all samples, thereby reducing noise while maintaining biologically meaningful richness estimates. Bacterial alpha diversity, including the Chao1, Shannon, Simpson, Observed and Fisher indices, was estimated using JMP software. The results of alpha diversity were visualized and statistical analysis were conducted using JMP software (JMP®, 2025). We performed alpha diversity analysis of bacterial communities in the leaf endosphere of maize using a nested analysis of variance (ANOVA) model that included plant group (teosinte, Mexican maize, US maize) and genotype (perennial teosinte, Balsas teosinte, Mexican landrace, Mexican elite inbred, US landrace, US elite inbred) nested within plant group. As warranted, after ANOVA plant group means were separated using Tukey’s tests; genotype means were compared using five *a priori* contrasts: perennial vs Balsas teosinte; Balsas teosinte vs Mexican landrace; Mexican landrace vs US landrace; Mexican landrace vs Mexican elite inbred; and US landrace vs US elite inbred. The critical *P* value for each *a priori* contrast was set to 0.010 to maintain the experiment-wise error rate at 0.05 (Abdi and Williams, 2010). To measure bacterial beta diversity, we used Principal Coordinate Analysis (PCoA) based on Bray-Curtis and weighted UniFrac metrics (Bray and Curtis, 1957; Lozupone et al., 2007). A nested permutational analysis of variance (PERMANOVA) was performed to assess the differences in community composition among plant group (teosinte, Mexican maize, US maize) and genotype (perennial teosinte, Balsas teosinte, Mexican landrace, Mexican elite inbred, US landrace, US elite inbred) nested within plant group. Pairwise comparisons of genotype were calculated using pairwise PERMANOVA function based on adjusted false discovery rate (FDR) P-values. Further, distance to centroid based on Bray-Curtis dissimilarity metric and Nonmetric Multidimensional Scaling (NMDS) analysis was conducted to test for differences in beta diversity between plant samples (Anderson, 2001). PCoA, centroid plot, and NMDS analyses were performed using “phyloseq”, “microbial “, “microeco”, “file2meco”, and “magrittr” R packages (microbial R package; Anderson, 2001; McMurdie and Holmes, 2013; Liu et al., 2021; Bache and Wickham, 2022; Liu et al., 2022). NMDS plots were visualized in R, while the PCoA and distance to centroid plots were visualized in JMP Pro 17 statistical software (JMP®, 2025) using output file form microeco package. Weighted UniFrac metric was calculated in Mothur using “unifrac.weighted” and visualized as PCoA plot in R. The output files from “pcoa” command line in Mothur were utilized in R generating the plot. R packages including “rgl”, “vegan”, “tidyverse”, “ggtext”, “ggplot2”, and “stats” were employed for visualization of Weighted UniFrac plot (Murdoch, 2001; Wickham, 2016; Wickham et al., 2019; Wilke, 2020; Oksanen et al., 2022; R Core Team, 2022). Cluster dendrogram based on Bray-Curtis similarity was calculated from OTUs with the “vegdist” function in R (Oksanen et al., 2022). Reductions in stringency and increases in variability of processes involved in microbial recruitment in leaf and AKP were assessed using the beta-nearest taxon index (βNTI). Phylogenetic distances between microbial communities were also evaluated using βNTI (Shade and Handelsman, 2012). The beta mean nearest-taxon distance (betaMNTD) was calculated using a ‘sample pool’ null model analysis with 999 randomizations within the picante package in R, and the results were visualized using the ggplot2 package to provide a comprehensive understanding of microbial community composition (Kembel et al., 2010; Wickham, 2016).

Linear discriminant analysis effect sizes (LEfSe) were used to determine differentially abundant features between plant types (Segata et al., 2011). LefSe was calculated by setting a *p-value* of less than 0.05 and the logarithmic LDA (linear discriminant analysis) effect size score threshold was set to 4.0. The LEfSe analysis was conducted using “phyloseq”, “microeco”, “file2meco”, and “ggplot2” R packages (McMurdie and Holmes, 2013; Wickham, 2016; Liu et al., 2021; Liu et al., 2022). The relative abundances of leaf endosphere microbiota at the phylum and genus levels were generated in R. UpSet plot and a Venn diagram, which shows unique or shared OTUs between genotypes, and were calculated in R using the “microeco” package (Liu et al., 2021). Shared OTUs were defined as core microbiome (Shade and Handelsman, 2012). Finally, the bacterial functional profiles of the six genotypes were determined through Functional Annotation of Prokaryotic Taxa (FAPROTAX) (Louca et al., 2016).

## Results

We sequenced the bacterial 16S rRNA V4 region from 83 leaf samples from 17 plant accessions corresponding to six genotypes spanning the evolution of maize from perennial teosinte to elite inbred maize. From these samples we obtained 3,921,857 bacterial sequences, which yielded 2,714,777 total sequences and 189,434 unique sequences after running “screen.seqs”, “unique.seqs” and “align.seqs” command lines. We reran the “screen.seqs” command, per the Mothur pipeline, to ensure that all the sequences aligned with the same region and obtained 2,545,257 total sequences and 156,886 unique sequences. We reran the “unique.seqs” step once more after quality filtering to eliminate duplicates, which yielded the same total number of sequences as the previous step but 151,799 unique sequences. After these steps, we ran chimera clustering and removal steps which yielded 2,490,998 total sequences and 73,348 unique sequences. Finally, we removed non-target taxonomic lineages using the “remove.lineage” command and obtained 99,623 total and 24,666 unique bacterial sequences. After rarefaction, we obtained 423 bacterial OTUs for further analysis.

**Figure 1:**
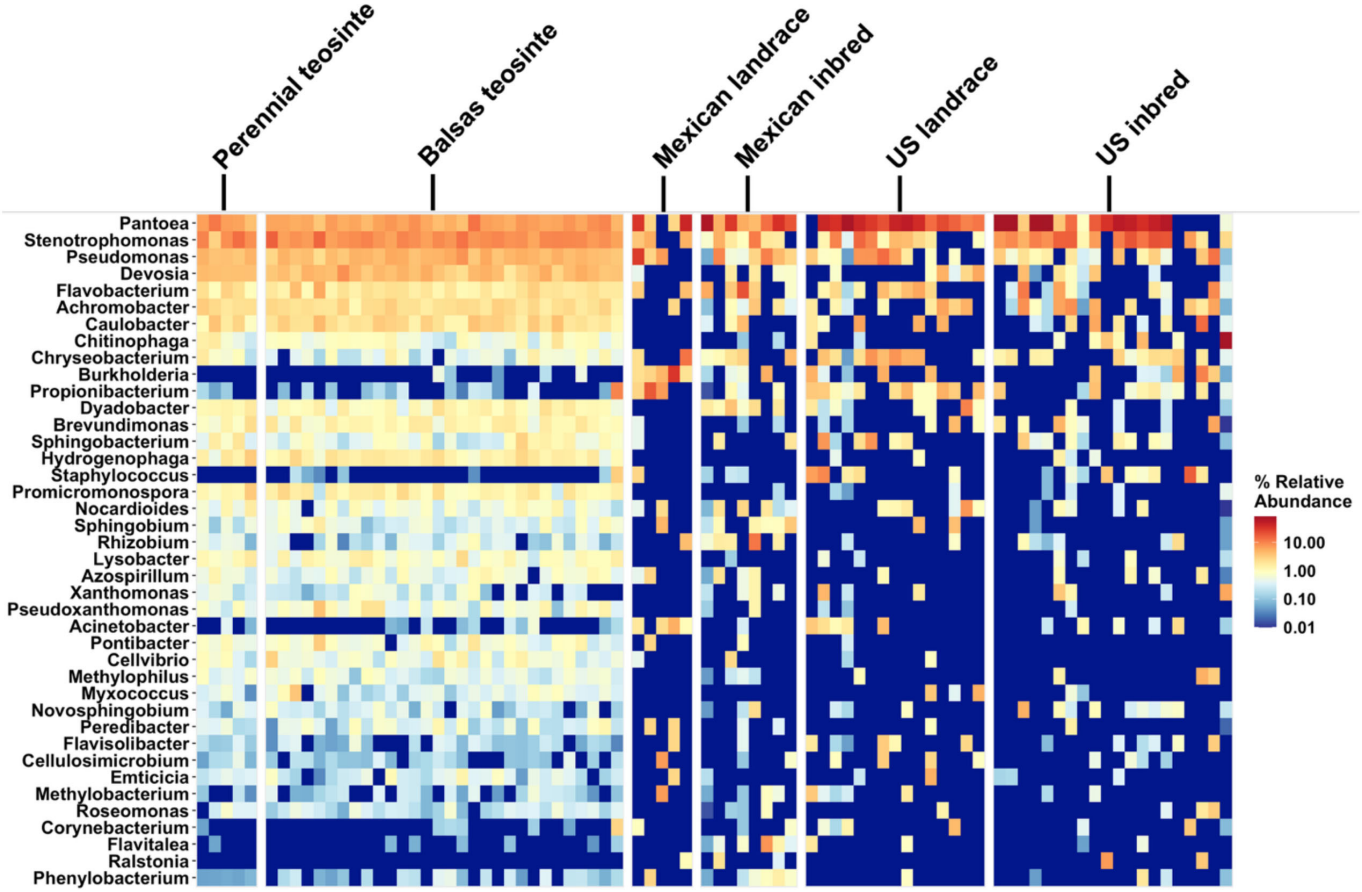
Heatmap illustrating the relative abundance of the top 40 taxa OTUs at genus-level in maize genotypes. The genotypes are ordered to span the evolution of maize: perennial teosinte, Balsas teosinte, Mexican landrace maize, Mexican inbred maize, US landrace maize, and US inbred maize, as shown at the top.

The OTUs fell into a total of 229 genera from 19 phyla, according to taxa similarities with sequences in the SILVA database. The most dominant taxa among the 40 most abundant (per relative abundance) bacterial genera were either lower in abundance or completely missing in the maize landraces and elite inbreds compared to the teosintes (**Figure 1**). Marked decreases in the relative abundances of the genera *Devosia, Caulobacter, Chitinophaga*, and *Dyadobacter*, among others, are evident from the teosintes to the maizes. A few genera seemed more abundant in maize compared to teosinte, such as *Pantoea, Staphylococcus, Acinetobacter, Corynebacterium*, and *Ralstonia* (**Figure 1**). At the phylum level, Proteobacteria (65.5%), Bacteroidetes (14.1%), Actinobacteria (9.26%), Firmicutes (3.40%), and TM7 (1.92%) were the most dominant phyla in maize leaf endosphere bacterial communities. Notably, the relative abundance of Actinobacteria was consistently high in the teosintes and varied between low and high in maize, suggesting a marked effect of domestication (**Supplementary Figure S1**).

**Figure 2:**
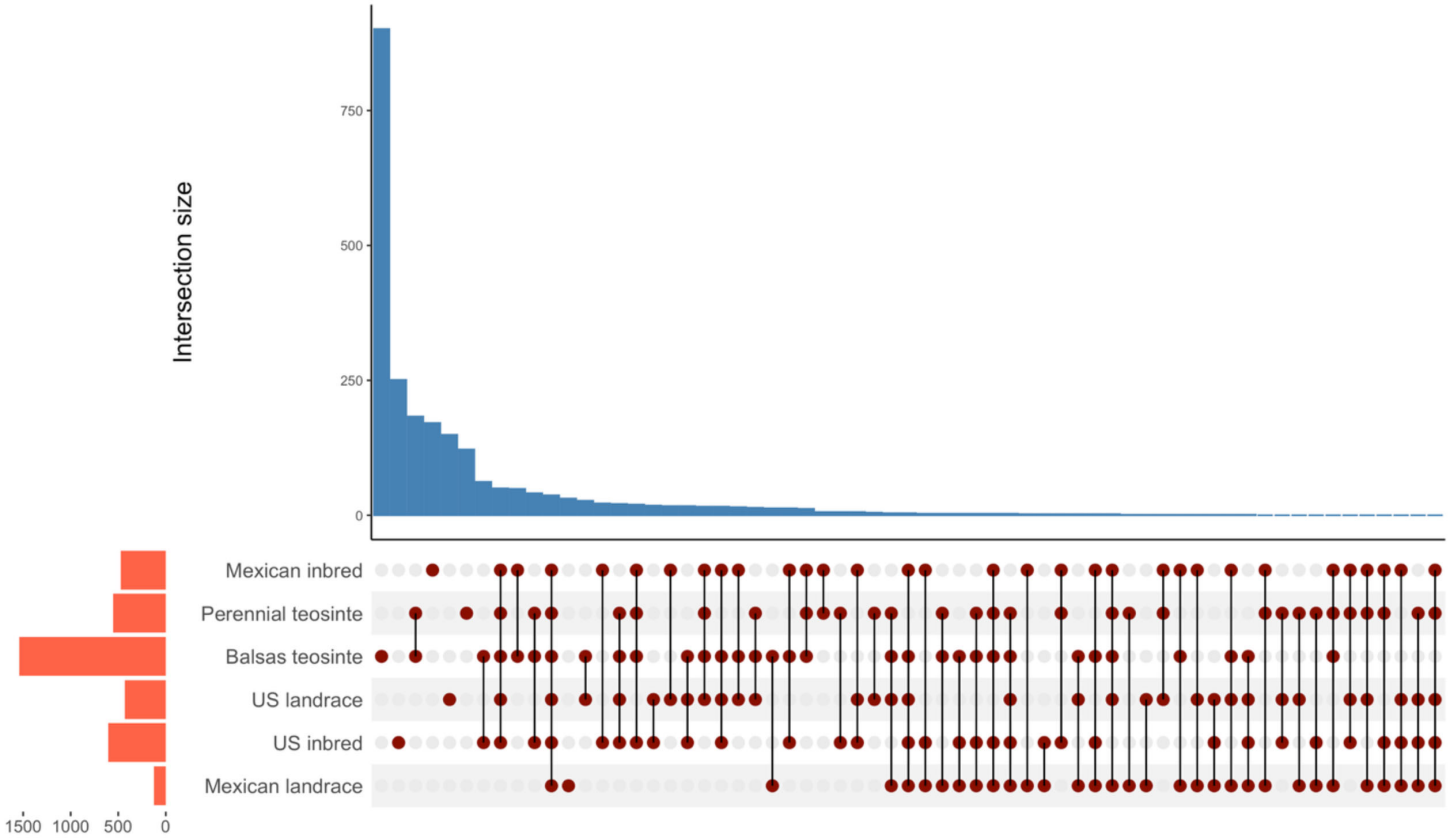
UpSet plot showing shared and unique bacterial OTUs between maize genotypes in the leaf endosphere microbiota. Perennial teosinte harbored 122 unique bacterial taxa, Balsas teosinte 901, US inbred 251, US landrace 149, Mexican inbred 171, and Mexican landrace 31, respectively.

The shared and unique OTUs among maize genotypes were assessed and visualized using UpSet plot (**Figure 2**). There were 37 OTUs shared by all plant genotypes, making them the central core microbial community shared by all the *Zea* genotypes studied here. Overall, the core microbial community of the six genotypes included 37 OTUs in 17 unique genera. Balsas teosinte and US inbred genotypes harbored the highest numbers of unique bacterial taxa, with 901 and 251, respectively, followed by US landrace with 149, Mexican inbred with 171, perennial teosinte with 122 and Mexican landrace with 31 unique taxa (**Supplementary Figure S2**).

**Figure 3:**
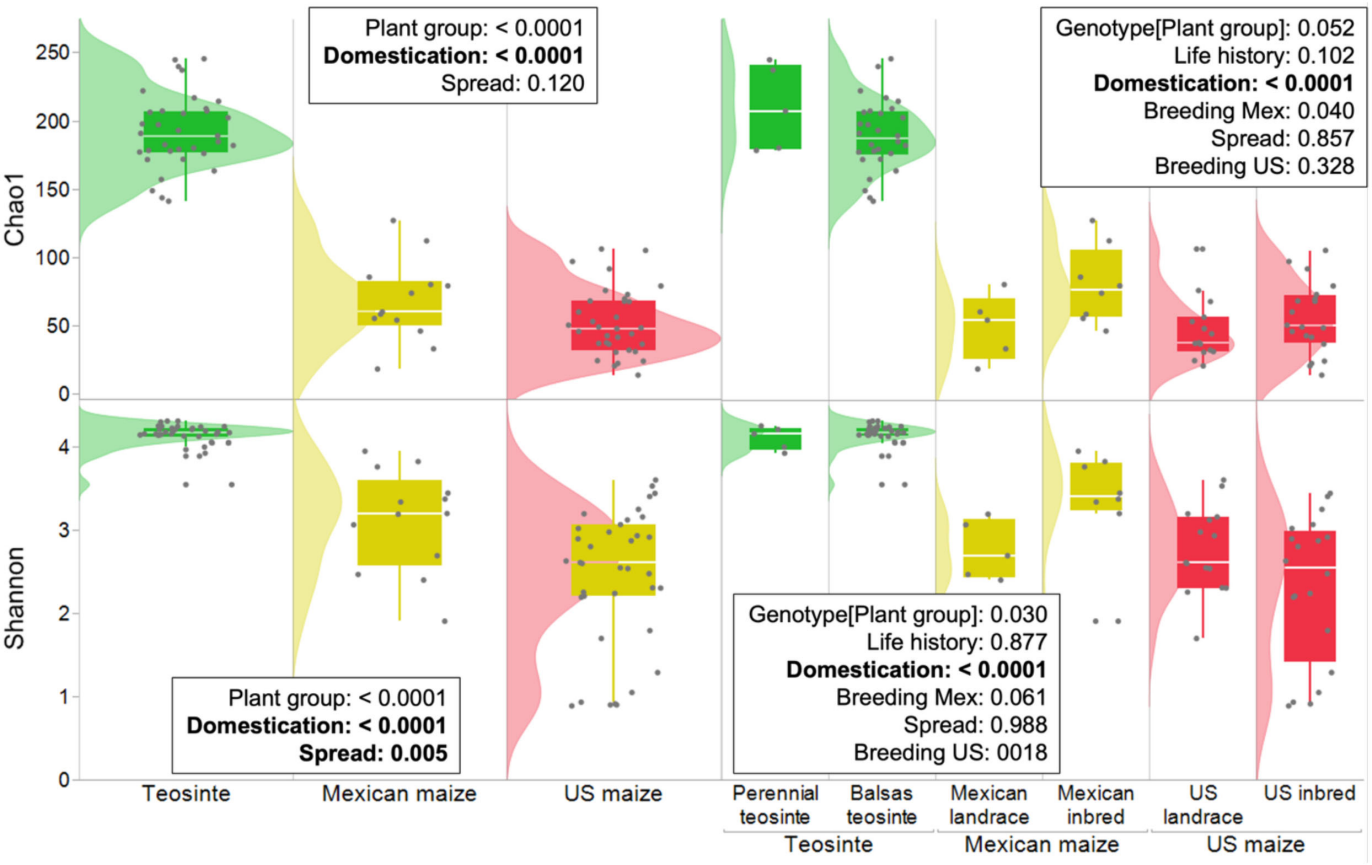
Box plots and histograms showing leaf endosphere alpha diversity indices observed in maize genotypes. In the left plot, Teosinte, Mexican maize, and US maize were compared using Tukey’s test. In the right plot, nested groups within Teosinte (perennial and Balsas), Mexican maize (landrace and inbred), and US maize (landrace and inbred) were compared using a-priori contrasts. The Shannon and Chao1 indices exhibit a pattern of decreasing diversity and richness from Teosinte to Mexican maize (landrace and elite inbred) and to US maize (landrace and elite inbred). Teosintes generally showed greater diversity and richness in the leaf endosphere microbiota (P<0.05).

The loss of diversity associated with domestication was particularly evident in the diversity indices associated with the plant groups and genotypes (**Figure 3**). Comparisons among Shannon and Chao1 indices of leaf endosphere microbiota among the plant groups revealed a trend of declining diversity and richness from teosinte to Mexican maize (landrace and elite inbred) and US maize (landrace and elite inbred), as well as a consistent decline between the Balsas teosinte and Mexican landrace genotypes, indicating an effect of domestication (P<0.0001, **Figure 3**). These findings were further supported by the richness OTUs, and Simpson and Fisher indices (**Supplementary Figure S3**). Breeding (i.e., landrace vs elite inbred maize comparison) did not have a consistent effect on OTU diversity as its effect was significant only per Shannon index and for Mexican maize (P = 0.005).

**Figure 4:**
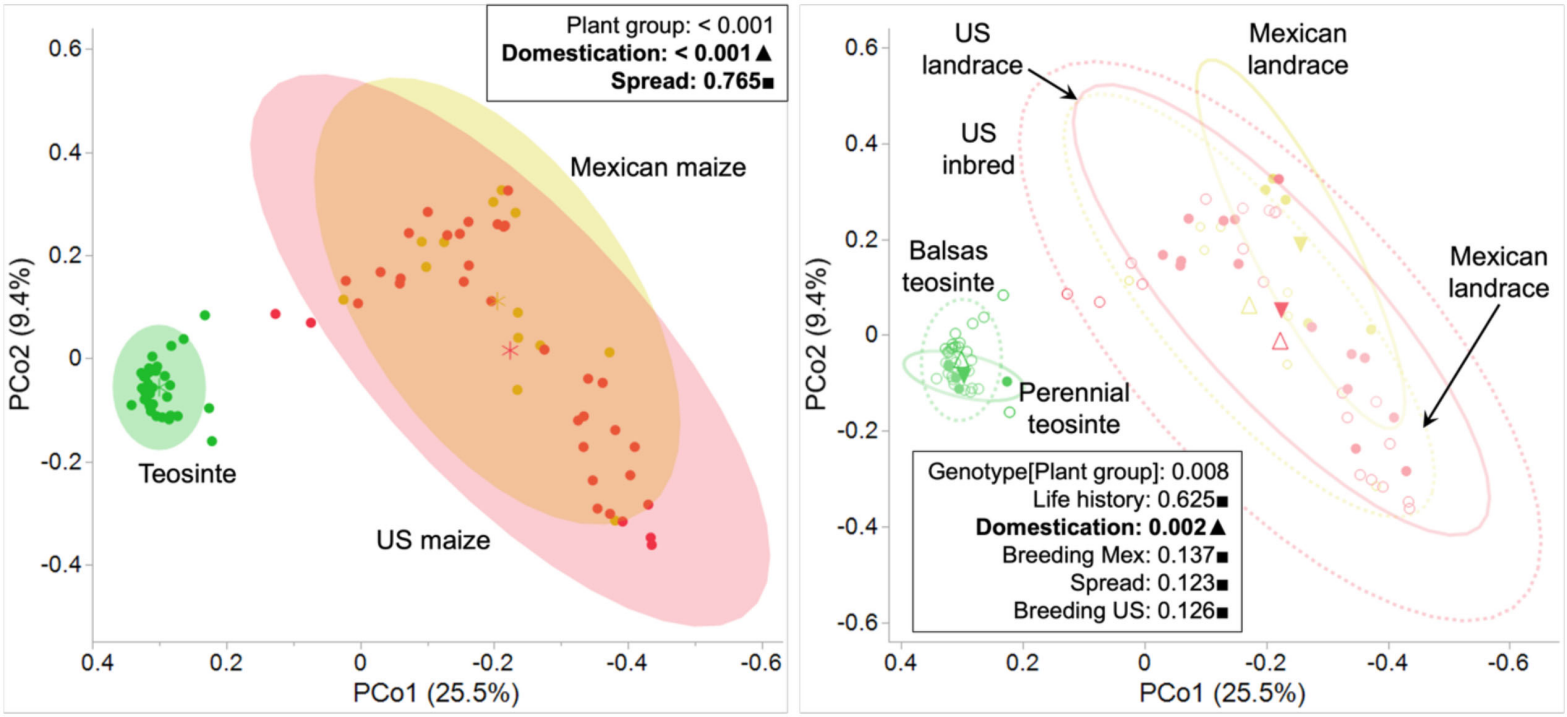
Leaf endosphere beta diversity is represented as a principal coordinates analysis (PCoA) plot based on Bray-Curtis dissimilarity values among maize genotypes. Each point represents a single sample, and the shape and colors indicate individual maize genotype. PCoA plot demonstrate significant differences in leaf endosphere microbiota composition between teosintes and maize plant group along the first axis (PCo 1, 25.5%), while differences along the second axis (PCo 2, 9.4%) were comparatively minor. The distinct pattern observed between teosintes and maize along the first axis indicated that domestication has influenced beta diversity of leaf endosphere bacterial communities. Centroids are represented by empty black symbols.

**Figure 5:**
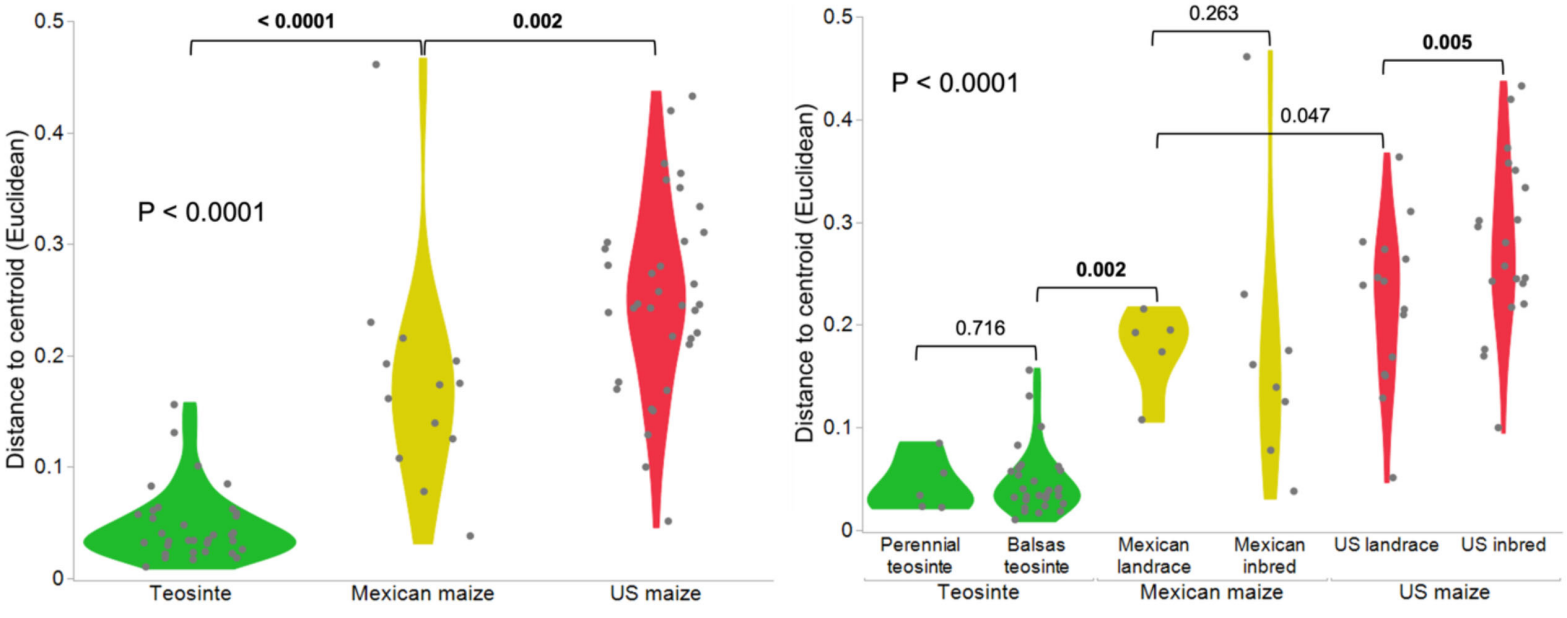
Violin plot illustrates distance to centroid based on Euclidean dissimilarity metric, demonstrating variability in maize genotypes across the effect of life history, domestication, northward spread, and breeding. The x-axis represents plant group and genotypes: Teosinte (perennial and Balsas teosinte), Mexican maize (Mexican landrace and inbred), and US maize (US landrace and inbred).

We used Principal Coordinates Analysis (PCoA) based on Bray-Curtis dissimilarity matrices of bacterial communities to analyze and visualize beta diversity dissimilarities among the six genotypes (**Figure 4**); corresponding weighted Unifrac metric and NMDS plots are shown in **Supplementary Figure S4**. PCoA revealed marked divergence in the composition of leaf endosphere microbiota between the teosinte and maize genotypes along the first axis (PCo 1, 25.5%), while little to no divergence was evident among genotypes along the second axis (PCo 2, 9.4%). The divergent pattern separating teosinte and maize along the first axis suggested a potential effect of domestication on beta diversity of leaf endosphere bacterial communities. Nested permutational analysis of variance (PERMANOVA) revealed significant effects of plant group (R^2^ = 0.2469, P < 0.001), whereas genotype nested within plant group was not significant (R^2^ = 0.04834, P = 0.008) (**Table 1A**). Comparisons among plant groups of leaf endosphere microbiota indicated significant effects of domestication in beta diversity (Teosinte vs Mexican maize, P = 0.001) while comparisons among genotypes revealed significant effects of domestication (Balsas teosinte vs. Mexican landrace, P = 0.002) (**Table 1B**). In addition, hierarchical clustering analysis based on the Bray-Curtis distance of bacterial OTUs from leaf samples showed clear separation between teosinte and maize samples (**Supplementary Figure S5**). Importantly, the within-cluster spread (= average distance to centroid) was significantly greater in maize, both Mexican and US, compared to teosinte (P < 0.001) (**Figure 5**). The increases in variation (within-cluster spread) with domestication are consistent with predictions of the Anna Karenina Principle, which predicts that under stress, microbial communities are increasingly shaped by stochastic processes and suffer overall increases in the differences between communities (Arnault et al., 2023).

**Table 1:** (A) Results of PERMANOVA analysis on leaf endosphere microbiota showed significant effects of plant group and nested genotype (plant group). (B) Pairwise comparisons of PERMANOVA based on adjusted false discovery rate (FDR) P-values identified significant differences among maize plant group and genotypes (FDR adjusted P<0.05).

(A)			
PERMANOVA results for plant group	R^2^	F	Pr (>F)
Plant group	0.2469	13.48	**0.001**
Genotype (Plant group)	0.04834	1.76	**0.008**
Residual	0.70475		
Total	1		

(B)			
Plant group comparison	R^2^	F	Pr (>F)
Teosinte vs. Mexican maize	0.81582	203.75	**0.001**
Mexican maize vs. US maize	0.008871	0.41	0.765
Genotype comparison	R^2^	F	P_adj_
Perennial teosinte vs. Balsas teosinte	0.02135	0.72	0.625
Balsas teosinte vs. Mexican landrace	0.81006	140.7	**0.002**
Mexican landrace vs. Mexican inbred	0. 18914	2.56	0.137
Mexican landrace vs. US landrace	0.11642	2.37	0.123
Us landrace vs. US inbred	0.06586	2.32	0.126

Significant values are indicated in bold.

**Figure 6:**
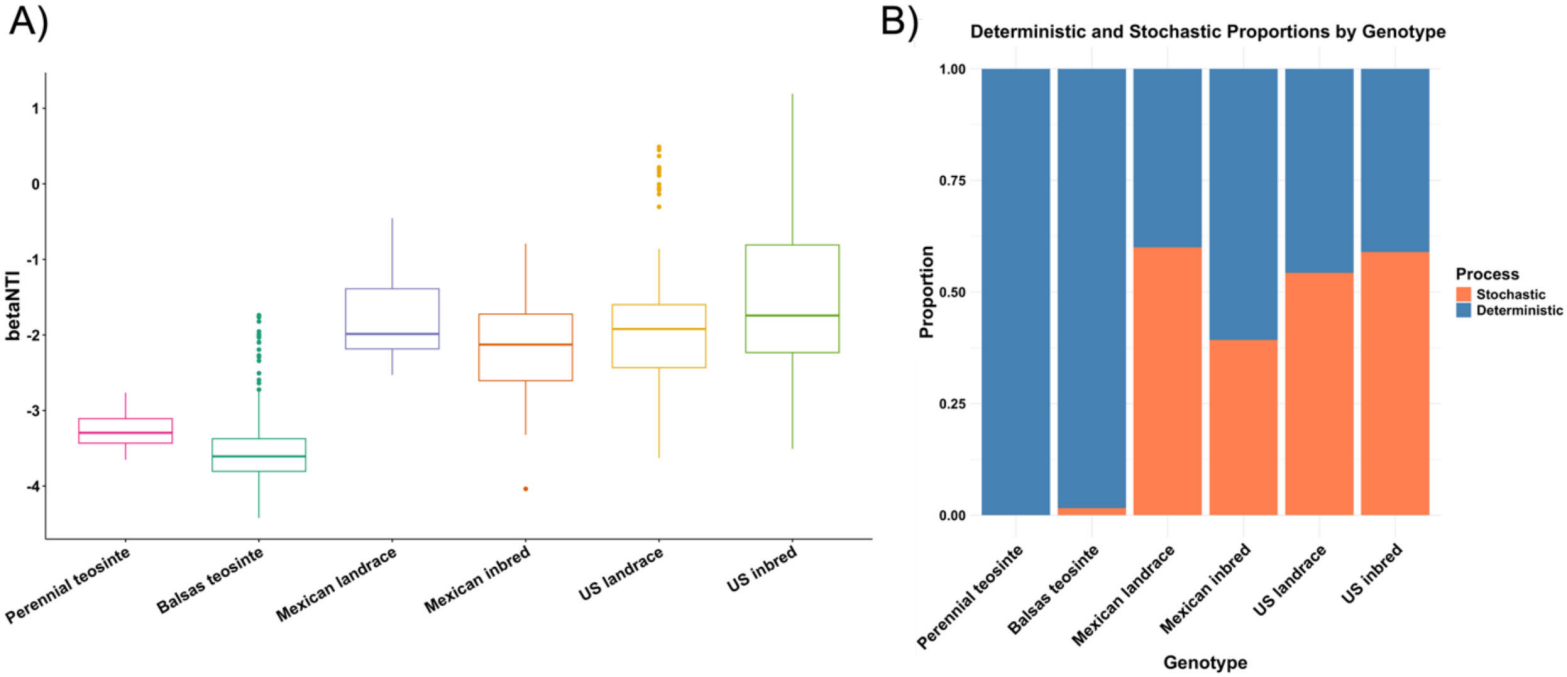
The boxplot illustrates the βNTI values across maize genotypes **(A)**, while the stacked bar plot shows the proportions of stochastic (orange) and deterministic (blue) processes contributing to community assembly within each genotype group **(B)**. Teosintes exhibited βNTI values < -2, indicating a stronger influence of deterministic processes. In contrast, Mexican and US maize genotypes displayed βNTI values between -2 and 2, suggesting that community assembly in these groups was primarily governed by stochastic processes, with a comparatively smaller contribution from deterministic factors.

The beta-nearest taxon index (βNTI) is a critical metric for elucidating the processes governing microbial community assembly, distinguishing between deterministic and stochastic influences. A βNTI value > 2 signifies that heterogeneous selection drives community composition, where varying environmental pressures across sites lead to greater phylogenetic divergence and reduced taxonomic overlap compared to random expectations. Conversely, a βNTI < -2 indicates homogeneous selection, wherein consistent environmental conditions promote phylogenetic convergence and tighter community clustering. Values of βNTI between -2 and 2 align with the null model, suggesting that stochastic processes, such as random dispersal, ecological drift, or historical contingencies, predominantly shape community composition. In this study, teosintes were found to have βNTI values < -2, indicating a dominance of deterministic processes, whereas Mexican and US maize genotypes exhibited βNTI values between -2 and 2, suggesting stochastic processes as the primary drivers of community assembly (**Figure 6A**). Furthermore, the proportions of deterministic and stochastic influences were quantified, revealing that deterministic processes had a stronger effect on teosintes, while Mexican and US maize genotypes were predominantly shaped by stochastic processes with a smaller contribution from deterministic factors (**Figure 6B**).

**Figure 7:**
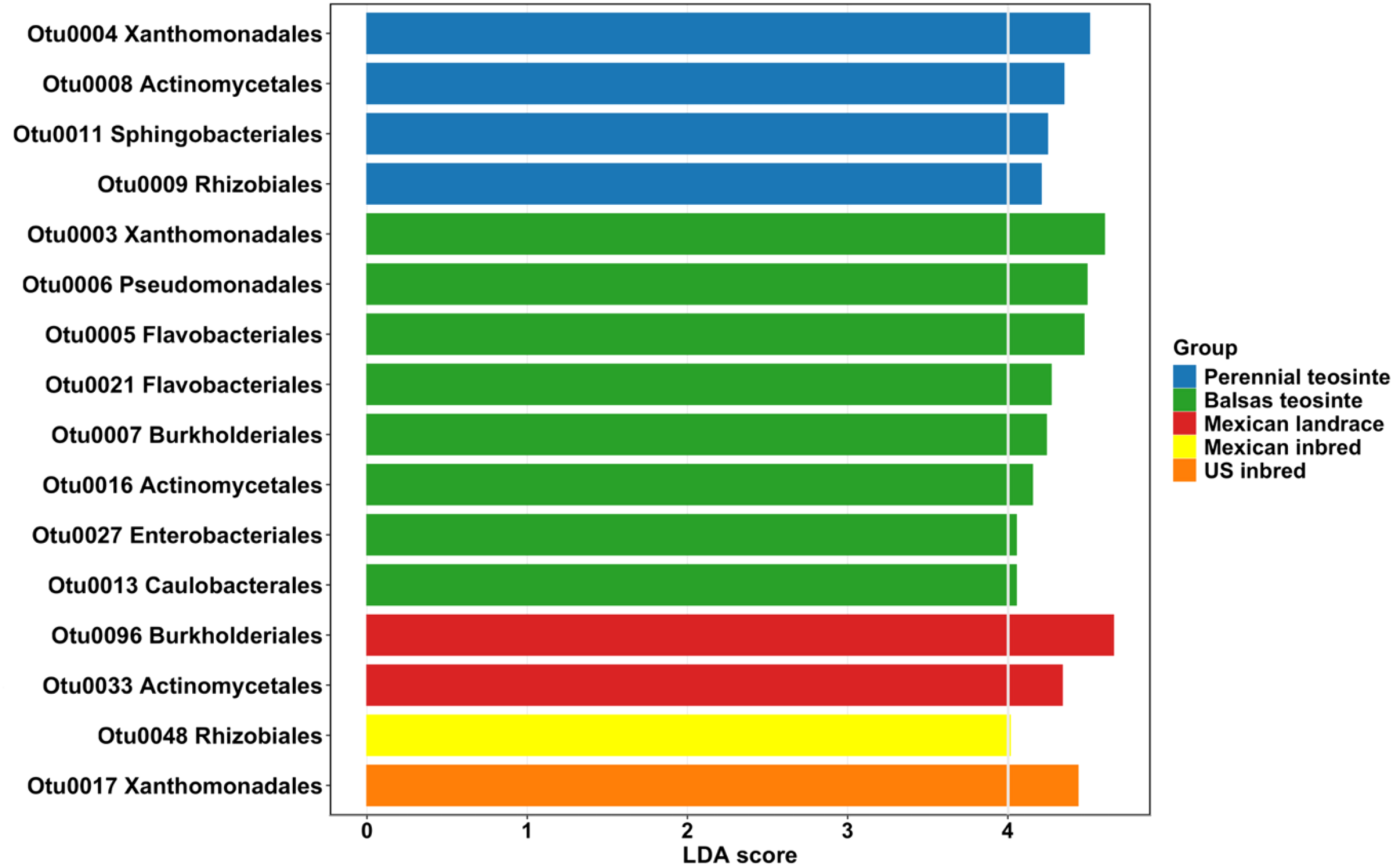
Linear discriminant analysis (LDA) effect size (LEfSe) analysis of biomarker taxa at order level among maize genotypes. Horizontal bars represent enriched taxa and the effect size of each taxon, and the colors indicate the genotype. LDA score cutoff 4 with an alpha value of 0.05 was used to distinguish bacterial taxon. LEfSe analysis indicates that the majority of differentially abundant taxa were more prevalent in the teosinte genotypes, including both perennial and Balsas varieties. Mexican inbred and US inbred line genotypes had one taxon at the order level. There were two orders in Mexican landrace. Balsas teosinte exhibited eight orders, while the perennial teosinte showed four.

The LDA effect sizes (LEfSe) analysis, with LDA score cutoff at 4 and alpha value of 0.05, was conducted to identify biomarker taxa at the order level. Single order exceeded the cutoff in the Mexican inbred line and US inbred line genotypes two orders in the Mexican landrace genotype, eight in the Balsas teosinte genotype, four in the perennial teosinte genotype, and none in the US landrace genotype (**Figure 7**). Comparisons of each genotype as a paired group were further analyzed to identify significantly enriched taxa based on the Kruskal-Wallis rank-sum test (α = 0.05) (**Supplementary Figure S6**). LEfSe analysis showed most of the differentially abundant taxa were enriched in the teosinte genotypes, both perennial and Balsas. In genotype-wise comparisons, most of the differentially abundant taxa were similarly enriched in the teosinte genotypes(**Supplementary Figure S6**).

**Figure 8:**
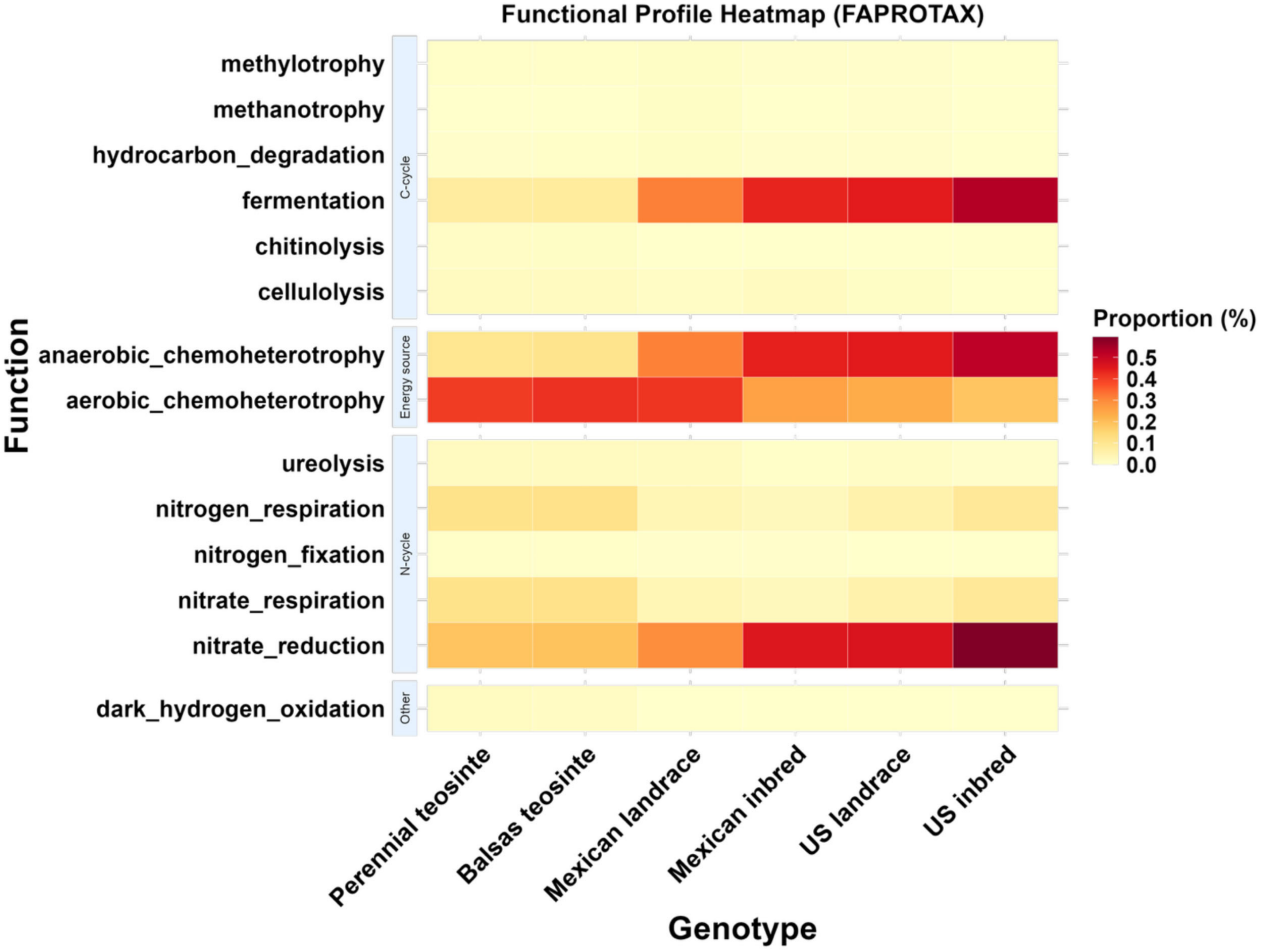
Functional Annotation of Prokaryotic Taxa (FAPROTAX) analysis of leaf endosphere of maize genotypes. FAPROTAX predictions indicated that the teosintes exhibited higher cellulolytic, chitinolytic, nitrogen respiration, and nitrate respiration functions, while maize landraces and elite inbreds displayed higher fermentation and nitrate reduction functions.

Lastly, FAPROTAX functional prediction analysis showed that the perennial and Balsas teosinte genotypes had higher nitrate and nitrogen respiration interactions than the maize genotypes (**Figure 8**). In contrast, the US inbred- and US landrace maize genotypes, followed by Mexican inbred and Mexican landrace genotypes had higher fermentation and nitrate reduction interactions compared to the teosinte genotypes (**Figure 8**).

## Discussion

In this study, we examined the leaf endosphere microbiota of a suite of six teosinte and maize (*Zea* spp.) genotypes spanning the evolution of maize from teosintes (perennial and Balsas teosinte) to maize landraces (Fr. Mexico and United States) and maize elite inbreds (Fr. Mexico and United States). Through comparisons among those six genotypes, we inferred on effects of transitioning from perennial to annual life history in the teosintes (perennial vs Balsas teosinte), domestication (Balsas teosinte vs Mexican landrace maize), northward spread (Mexican vs US landrace maize), and breeding (Mexican and US landrace vs Mexican and US inbred maize). In line with Anna Karenina principle predictions (Zaneveld et al., 2017; Chen et al., 2020; Arnault et al., 2023), we expected that teosinte’s transition from perennial to annual life history, and maize domestication, northward spread, and breeding would be associated with increasing variability in bacterial communities driven by stochastic processes. Importantly, we found that domestication in particular, evident both in comparisons between the Balsas teosinte and Mexican landrace genotypes, as well as between teosintes and maizes broadly, was associated with decreasing leaf endosphere bacterial diversity and increasing variability in bacterial communities. This suggested that dysbiosis is associated with maize domestication. Below, we discuss our results showing that maize domestication significantly affected α-diversity and β-diversity of the leaf endosphere microbial community, and other differences in the structure and assemblage of the leaf endosphere microbial community associated with maize domestication, northward spread, and breeding.

### Maize domestication significantly affected α-diversity and β-diversity of leaf endosphere microbial community

We found that the Shannon and Chao1 diversity values in the Balsas teosinte genotype were higher than in the Mexican landrace genotype, and that generally they were higher in the teosintes compared to the maizes. We propose that the reductions are due to an increasing dominance of stochastic over deterministic processes mediating the leaf microbiome’s assemblage. The diversity decreases both in Mexican landrace maize and maizes combined relative to their predecessors indicate that the decreases are associated with domestication, and in parallel with a transition from plant survival and reproduction in a highly variable natural environment to a typically richer and more predictable agricultural environment (Bernal and Medina, 2018; Fontes-Puebla and Bernal, 2020). In contrast, there were no significant changes in diversity associated with the transition from perennial to annual life history, northward maize spread, and breeding. To our knowledge, ours is the first study reporting a significant decay in leaf endosphere bacterial diversity and richness associated with maize domestication. Importantly, our study reveals significantly higher bacterial diversity and richness in the leaf endosphere of Balsas teosintes compared to the maize inbreds that serve as parents of commercial hybrid varieties, while highlighting a decline in bacterial diversity with likely implications for maize productivity in environments under stress from climate change.

Our PCoA analysis revealed a clear divergence between teosinte and maize in variation, in addition to diversity, associated with their leaf bacterial communities. In addition, our study highlighted distinct patterns in the clustering of teosintes, including perennial and Balsas teosinte, compared to the more variable distributions observed in maize landraces and elite inbreds. Notably, the beta diversity in leaf bacterial communities exhibited variations among plant groups, with teosinte displaying higher diversity in the leaf endosphere than the maize groups. Consistently, a previous study on the microbiome of wild and domesticated wheat species showed that the bacterial communities in the leaves of wild wheat species were more phylogenetically clustered compared to the bacterial communities in domesticated wheat (Hassani et al., 2020). In terms of the maize leaf endosphere microbiota study, previous studies have demonstrated no significant differences in the beta diversity of leaf-associated bacterial assemblages among modern maize cultivars (Kong et al., 2020; Wagner et al., 2020b). This is consistent with studies showing that host plants play important roles in shaping their endophytic bacteria communities (Agler et al., 2016; Wagner et al., 2020b; Xiong et al., 2021b; Singh et al., 2023). Thus, variation in the diversity between teosinte and maize plant groups in this study can be attributed to the selectiveness of the host plant. In addition, such selection may be correlated with host plant functional traits and ecological strategies (Kembel et al., 2014; Laforest-Lapointe et al., 2017). Any underlying mechanisms and consequences of diminished selection effect remain to be explored.

Collectively, we observed increased beta-diversity and decreased alpha-diversity in leaf-associated bacterial communities coincident with maize domestication. This pattern is observed both in comparisons between Balsas teosinte and Mexican landrace genotypes, as well as more broadly between teosintes and maizes. This is potentially due to decreased selection and increased relevance of stochastic processes. Patterns similar to these are also noted in association with stress in plants and other hosts, including humans and animals (Turnbaugh et al., 2009; Abrahamsson et al., 2014; Zaneveld et al., 2017; Rocca et al., 2019; Lavrinienko et al., 2020). For instance, mutations of immunity-related genes in *Arabidopsis*, and disease in Korean fir and chili pepper were associated with reductions in the diversity of their microbial communities (Chen et al., 2020; Gao et al., 2021; Han et al., 2022). Seemingly, hosts lose beneficial microbiota due to stochastic processes associated with stress. Additionally, the negative effects of stresses are compounded by dysbiosis, following Anna Karenina Principle predictions (AKP) (Petersen and Round, 2014; Vangay et al., 2015; Levy et al., 2017). AKP suggests a rise of stochastic over deterministic processes mediating microbial community composition within the holobiont (Zaneveld et al., 2017; Ahmed et al., 2019; Arnault et al., 2023). We found that teosintes exhibited βNTI values < -2, indicating a stronger influence of deterministic processes, whereas Mexican and US maize genotypes showed βNTI values between -2 and 2, highlighting the predominance of stochastic processes with a smaller contribution from deterministic factors. The proportions of deterministic and stochastic influences further supported these observations. The decline in diversity and increase in variability in the bacterial community of the maize leaf endosphere observed in this study reveal patterns suggestive of dysbiosis. The increased variability within the bacterial community is consistent with AKP. We suggest that the maize leaf endosphere exhibits patterns akin to dysbiosis. The decline in the diversity of microbial species in leaf endosphere of the maizes compared with the teosintes may impact the crop’s ability to cope with biotic and abiotic stresses (Gutierrez and Grillo, 2022; Alam and Purugganan, 2024), particularly as environments change rapidly under climate change. Although we identified microbial signatures of dysbiosis through AKP, we did not evaluate the functions of the leaf endosphere microbiota in host fitness in this study. These results provide a basis for future investigations into the effects of leaf endosphere microbial functions in teosintes and maize on host fitness.

Current knowledge of plant microbiome dysbiosis, although limited, is largely derived from studies of pathogenic plant states. However, evidence also indicates that dysbiosis can result from host genetic disruptions that impair microbiota homeostasis, such as loss of TIP1 function, environmental stressors, and agricultural practices. Cheng et al. (2024) identified TIP GROWTH DEFECTIVE 1 (TIP1) as a key regulator of leaf microbiota homeostasis in *Arabidopsis*. Loss of TIP1 function under high-humidity conditions led to dysbiosis, marked by a >1,000-fold increase in endophytic bacterial load, reduced microbial diversity, and dominance of *Pseudomonas* and *Stenotrophomonas*, while beneficial taxa such as *Bacillus* and *Paenibacillus* were nearly eliminated. This microbial imbalance was associated with chlorosis, tissue lesions, and constitutive activation of immune genes, consistent with an autoimmune-like phenotype. These symptoms were absent under axenic conditions, indicating that the immune responses were microbiota dependent. Transfer of the dysbiotic microbiota from tip1 plants to healthy plants induced disease-like symptoms, demonstrating that the altered microbial community alone was sufficient to cause tissue damage. Together, these findings show that disruption of host control over the leaf microbiota results in dysbiosis-associated autoimmunity, linking microbial imbalance to immune dysfunction in plants. Moreover, Wasimuddin et al. (2025) reported that chemical stressors, including arsenic and the herbicide terbuthylazine, induced dysbiosis in soil microbiomes. Exposure to these compounds significantly reduced bacterial richness and diversity, indicating a loss of microbial balance. Community composition shifted toward stress-tolerant taxa such as *Flavobacteriaceae*, *Burkholderiaceae*, and *Xanthomonadaceae*, suggesting functional reorganization under chemical stress. Soil microbial interaction networks were also altered, reflecting changes in community structure and stability. Collectively, these findings demonstrate that chemical stressors drive dysbiosis in soil microbiomes. In another study, Darriaut et al. (2024) demonstrated that grapevine decline was associated with microbial dysbiosis, characterized by shifts in the composition, diversity, and functional potential of belowground microbiomes across bulk soil, rhizosphere, and root endosphere compartments. Symptomatic vines exhibited reduced microbial richness, enrichment of stress-tolerant and potentially pathogenic taxa, and decreased mycorrhizal colonization, indicating a loss of microbial balance. The authors suggested that long-term soil management practices and the resulting decline in soil microbial resilience likely contributed to this dysbiosis, promoting the dominance of stress-adapted and pathogenic microorganisms over beneficial symbionts.

Teosintes defend against herbivorous insects and pathogens by a variety of means (Rosenthal and Welter, 1995; Rosenthal and Dirzo, 1997; Takahashi et al., 2012; Szczepaniec et al., 2013; Chavan, 2014; de Lange et al., 2014; Bernal et al., 2015), and differences in defense strengths and strategies between teosinte and maize seem to be associated with their divergent environments, i.e. typically poorer, wild environments for the former and richer, agricultural environments for the latter (Fontes-Puebla and Bernal, 2020; Fontes-Puebla et al., 2021; Bernal et al., 2023). Examining the diversity of leaf endosphere microbiota in wild crop relatives may provide insights to how traits that allow plants to survive in the wild can, alongside other enhancements, be utilized to improve the breeding process. Previous studies revealed effects of maize host genetics and environmental conditions on how domestication and breeding shaped rhizosphere-associated microbiomes. Brisson et al. (2019) demonstrated that hybrid breeding significantly altered rhizosphere microbial communities, with inbred maize lines harboring communities more similar to teosintes than to modern hybrids under nutrient-depleted soils. Barnes et al. (2024) further reported that root microbiomes differed between teosintes and modern maize, and that teosinte accessions from distinct native environments harbored unique microbial groups associated with temperature and elevation. Huang et al. (2022) showed that domestication and genetic improvement increased rhizobacterial diversity and modified network structure, with inbreds exhibiting greater modularity than teosintes and landraces. In contrast to these belowground findings, our analysis of leaf endophytic communities indicated that domestication was associated with reduced bacterial diversity and greater variability in community composition in the endosphere of maize landraces and inbreds relative to teosintes. Collectively, these results suggest that while domestication and breeding often enhanced microbial diversity and functional adaptability in the rhizosphere, they simultaneously imposed losses in stability in the leaf endosphere, providing the first evidence of endophytic dysbiosis associated with crop domestication.

### Differences in the structure and assemblage of leaf endosphere microbial community associated with maize domestication, northward spread, and breeding

We found that Bacteroidetes and Actinobacteria (both Proteobacteria) were the most dominant taxa in the leaf endosphere bacterial communities. Similar compositions have been found in studies of different plant varieties such as the phyllosphere microbiome of sorghum (Sun et al., 2021), leaf endosphere of prairie plants (Ding and Melcher, 2016), and leaf microbiota of *Arabidopsis* (Bodenhausen et al., 2013). Furthermore, we observed a decline in the relative abundance of several taxa from the teosinte group, including perennial and Balsas teosinte, to the maize inbred lines, including *Devosia* and *Caulobacter* (Proteobacteria), and *Stenotrophomonas and Pseudomonas* (Pseudomonadota). Moreover, five genera showed higher abundance in maize plant group, *Pantoea, Staphylococcus, Acinetobacter, Corynebacterium, Ralstonia*. These results are consistent with those of previous research (Wagner et al., 2020a) which identified *Pantoea* spp. as the dominant taxa in hybrid maize cultivars at an early growth stage. Similarly, our study highlighted *Pantoea and Ralstonia* as dominant taxa in leaf samples from elite inbred lines, including lines from Mexico and US. Additionally, *Staphylococcus* and *Corynebacterium* were also among the top 20 genera in the relative abundance analysis of that research (Wagner et al., 2020a). Interestingly, the depleted genera observed in elite inbred maize in our study were also not detected in the previous research. Previous studies have demonstrated that taxa within these genera can exhibit either plant growth-promoting or biocontrol functions, or act as plant pathogens. For instance, *Pantoea agglomerans* has been shown to promote plant growth (Luziatelli et al., 2020), whereas *Pantoea stewartii* subsp. *stewartii* is the causal agent of Stewart’s wilt disease in maize (Farthing et al., 2025). The functional roles of these taxa enriched in maize may therefore differentially influence host plant performance. Future studies should aim to classify maize-enriched endophytic leaf taxa at the species level to distinguish beneficial from pathogenic bacteria and to experimentally assess their functional effects on the host plant.

We identified a few OTUs as known beneficial bacteria, though we did not test their functional properties. For example, we identified *Methylobacterium* spp., which are well known phyllosphere colonizers with documented beneficial effects, e.g., production of phytohormones, and enhancement of seed germination and plant growth (Omer et al., 2004; Madhaiyan et al., 2005; Abanda-Nkpwatt et al., 2006; Palberg et al., 2022). Previous studies consistently reported Methylobacteriaceae as the most abundant or as a biomarker taxon for leaf microbiota studies in maize (Wallace et al., 2018; Xueliang et al., 2020; Xiong et al., 2021b). In our study, we did not observe Methylobacteriaceae as an indicator taxon among genotypes in LEfSe analysis, though they were found to be more abundant in the teosinte plant group than in Mexican and US elite inbred genotypes. Moreover, we identified that classes Xanthomonadales, Actinomycetales, Burkholderiales, Rhizobiales were enriched in the teosinte group and Mexican landrace genotype as potential biomarkers with different abundances. This is in line with Xiong et al. (2021a) who found Actinobacteria, Burkholderiaceae, and Rhizobiaceae to be abundant in the phylloplane and rhizosphere of maize during the seedling stage, even if they were not identified as biomarker taxa. Many strains within those taxa establish beneficial partnerships with their host plants, including biological nitrogen fixation, plant growth stimulation, and protection against plant pathogens (Conn et al., 2008; Erlacher et al., 2015; Alvarez et al., 2017; Wahyudi et al., 2019; Jaiswal et al., 2021). Our findings demonstrated that biomarker taxa of teosinte were significantly more enriched in that plant group when compared to elite inbred lines, suggesting that wild ancestors may harbor greater diversity of beneficial taxa than crops. Such enriched bacterial taxa may confer functional advantages to their host plants, including increased tolerance of biotic and abiotic stress and greater adaptability to new environments. Further study is needed to better understand the correlations and functions of these taxa in crop wild ancestors, as well as for harnessing them to improve plant growth and health in crops.

We found that the core microbiome consists of 36 taxa shared across six genotypes. The predominant classes within this central core microbiome were Alphaproteobacteria, Gammaproteobacteria, Actinobacteria, and Betaproteobacteria. The taxonomic affiliations observed in our findings are similar to those in other crop studies on the core microbiome of phyllosphere bacterial communities, e.g., tree leaves (Kembel et al., 2014), grasses (Grady et al., 2019), and *Arabidopsis thaliana* (Bodenhausen et al., 2013). Moreover, Johnston-Monje and Raizada (2011) detected a core microbiota of endophytes that remained conserved in *Zea* seeds. Core microbial communities establish enduring relationships with plant hosts and crucially influence biological processes of their host plants (Tian et al., 2017; Stopnisek and Shade, 2021; Zhang et al., 2022). Our finding provides insight into core bacteriome taxa in the leaf endosphere as shaped by maize domestication and breeding. We suggest that a core bacterial community potentially coexists in mutual syntrophy, which provided a reproducible and conserved suite of taxa during maize domestication and breeding. Further studies are needed to understand the functions of the core bacterial community in relation to the biological functions of the maize host plant.

We used FAPROTAX analyses to evaluate potential functions of the microbiota of the different plant genotypes. While not conclusive, results from these analyses are useful for indicating future research directions concerning the functional ecology of endophytic bacteria and their host plants. Ecological functions and functional abundance of microbial communities can vary with plant type, plant development, and environment, among other variables (Zhou et al., 2020; Xiong et al., 2021b; Cheng et al., 2022; Liu et al., 2022). Regulation of functional groups and shifts in functional groups indicate that plant-recruited microbes reflect the current needs of the host plant (Zhou et al., 2020; Cheng et al., 2022). The results of FAPROTAX suggested that nitrate reduction, nitrate respiration, fermentation, and cellulolytic activities were most prominent in the two teosinte genotypes, while nitrate reduction and fermentation were prominent among the four maize genotypes. The latter results align with findings from a previous study on maize hybrids (Xiong et al., 2021b). Our findings provide predicted potential functions of the active microbial community in leaf endosphere. However, experimental research is essential to validate and confirm the predicted functional roles of these bacteria in enhancing plant fitness.

In addition to genotype, plant developmental stage and age affect phyllosphere microorganisms through the release of specific hormones and biologically active compounds. Previous research has shown that plant phenology is a major factor influencing the assembly of both phyllosphere and rhizosphere microbiomes (Wagner et al., 2016; Manching et al., 2018; Walters et al., 2018; Xiong et al., 2021a). Chaparro et al. (2014) reported that plants are able to select particular groups of microbes at different stages of development and suggested that this process is mediated by the secretion of distinct mixtures of compounds and phytochemicals in root exudates that vary across developmental stages, thereby contributing to the recruitment of the rhizosphere microbiome. Further, Wagner et al. (2016) showed that both leaf and root microbiome composition changes with plant age, especially during early to mid-vegetative growth. Thus, a limitation of the present study is that the impact of leaf ontogeny on leaf endophytic bacterial communities in teosinte and maize was not evaluated. Leaves from each genotype were collected at a single developmental stage, namely the five-week vegetative stage, when plants were approximately at the V4-V5 growth stage with four to six fully collared leaves. Leaf ontogeny was not considered, as accurately determining the ontogenetic stage of individual leaves is challenging. Consequently, this study could not assess changes in leaf endophytic bacterial community composition across different stages of leaf ontogeny in teosinte and maize. Future studies using time-series sampling across multiple developmental stages and evaluating the effects of plant ontogeny on microbial recruitment would provide insight into the temporal dynamics of endophytic microbiome assembly.

The environment in which experiments are conducted is an important determinant of microbiome assembly. Greenhouse and field experiments can yield different outcomes in microbiome studies because multiple environmental factors influence microbial community composition, and microbial assemblages are known to shift under conventional agricultural conditions (Wagner et al., 2016; Favela et al., 2024). Because this study focused on host genotype effects on the leaf endosphere microbiota while minimizing environmental variation, experiments were conducted under controlled greenhouse conditions. Consequently, the findings cannot be extrapolated to absolute microbiome status under natural field conditions. Future studies should examine leaf endosphere microbiota of teosintes and maize in natural habitats, including the effects of environmental factors on these communities.

## Conclusion

The findings presented here suggest that maize domestication played a pivotal role in shaping the assembly of maize leaf endophytes, with the plant genotype being a primary driver of this assembly. This was particularly evident in our comparisons between Balsas teosinte and Mexican landrace maize. Indeed, those comparisons showed significant declines of microbial diversity in the leaf endosphere associated with maize domestication. Strikingly, we found a signature of microbial dysbiosis is also associated with maize domestication. Particularly, a shift in microbial community structure from highly stringent and regulated in Balsas teosinte to loose and unregulated in maize, especially in Mexican landrace maize, the immediate descendant of Balsas teosinte. Taken together, these results are in line with and add support to the Anna Karenina principle in microbial dysbiosis and represent the first evidence of microbial signatures of dysbiosis caused by plant domestication. Also, our study demonstrated that teosintes harbor a greater number of biomarker taxa than maize landraces and elite inbred cultivars. Collectively, our results suggested that the leaf endophytic bacterial assembly in maize was markedly influenced by its domestication.

Altogether, our study contributes to the characterization of the leaf-associated endophytic microbiota composition of maize wild ancestors, landraces, and elite inbred lines. The insights from our research set the stage for advancements in biological control, biofertilizer technologies, and maize breeding strategies, particularly through the examination of microbiomes of wild relative genotypes. Identifying beneficial bacterial microbes and understanding their functions is essential for developing microbial technologies for enhancing the sustainability and resilience of agricultural practices.

## Data Availability

The datasets presented in this study can be found in online repositories. The names of the repository/repositories and accession number(s) can be found below: https://www.ncbi.nlm.nih.gov/bioproject/PRJNA1137996.
