# Supplementary material for: Maize leaf endosphere microbiome was affected by domestication and shows patterns consistent with microbial dysbiosis
Source: Front Microbiomes. 2026 Feb 23;5:1735358. doi: 10.3389/frmbi.2026.1735358 (PMC12993669; doi:10.3389/frmbi.2026.1735358)
Supplement: Supplementary file 1 [file SupplementaryFile1.docx]

**Supplementary Information**

Maize Leaf Endosphere Microbiome Was Affected by Domestication and Shows Patterns Consistent with Microbial Dysbiosis

Ilksen Topcu^1^, Julio S Bernal^2^ & Sanjay Antony-Babu^1^

^1^Department of Plant Pathology and Microbiology, Texas A&M University, College Station, TX

^2^Department of Entomology, Texas A&M University, College Station, TX

Table S1 Maize genotypes

| Maize Genotypes | Origin | Reference* |
| --- | --- | --- |
| Perennial teosinte | Jalisco state, Mexico: |  |
| Corralitos | Ahuacapán, Autlán (19°36'58"N 104°18'21"W) |  |
| Balsas teosinte | Jalisco state, Mexico: |  |
| Talpitita | Talpitita, Villa Purificación (19°42’N, 104°48’W) |  |
| Los Naranjos | Los Naranjos de Abajo, Ejutla (19°55'27"N 104°04'54"W) |  |
| El Cuyotomate | El Cuyotomate, Ejutla (19°58’N, 104°04’W) |  |
| El Rodeo | El Rodeo, Tolimán (19°33’N, 104°03’W) |  |
| Las Raíces | Las Raíces, Ameca (20°36'21"N 104°13'08"W) |  |
| Guachinango | Guachinango, Ameca (20°35'24"N 104°22'51"W) |  |
| Mexican Landraces | Veracruz state, México: |  |
| Tuxpeño | El Palmar de Susana, Tecolutla (20°20′35.32″N, 96°53′25.42″W) | PI 511649 |
| Mexican inbred lines | México: |  |
| CML277 | Ciudad de México, Mexico- Iowa, United States | PI 692142 |
| CML103 | Ciudad de México, Mexico- Iowa, United States | PI 692139 |
| US Landraces | United States: |  |
| Gourdseed | Ennis, Texas | PI 414179 |
| Lancaster Sure Crop | Ohio | PI 280061 |
| Reid Yellow Dent | Indiana | PI 213698 |
| US inbred lines | United States: |  |
| B73 | Iowa | PI 550473 |
| Hp301 | Indiana | PI 587131 |
| Mo17 | Missouri | PI 558532 |
| W438 | Wisconsin | AMES 29447 |

*USDA, ARS GRIN reference number

**Method S1** Detailed description of DNA extraction using ZymoBIOMICS™ DNA Miniprep Kit (Zymo Research, Irvine, CA, USA) and modified protocol;

Leaf samples were cut into small pieces (~1cm) and placed into a ZR BashingBead^TM^ Lysis Tubes to which 750 μl ZymoBIOMICS Lysis Solution was added. The tubes were then fitted to a bead beater (Fisherbrand^TM^ Bead Mill 24 Homogenizer, Fisher Scientific, USA) and processed for one minute at 2.40m/s speed. The lysed samples were centrifuged at 10,000 x *g* for 1 minute, and 400 μl aliquots of the supernatant were transferred to the Zymo-Spin III-F Filter in a collection tube. The lysate was bound to the columns by spinning at 8,000 x *g* for 1 minute and the flowthrough collected. Following this step, 800 μl of ZymoBIOMICS DNA Binding Buffer and 400 μl of 95% ethanol were added to the filtrate in the collection tube. Subsequently, 800 μl of the mixture from collection tube was transferred to a Zymo-Spin IICR Column in a collection tube and centrifuged at 10,000 x *g* for 1 minute. Flowthrough from the tube was collected and the process repeated with the addition of 800 μl of buffer and 400 μl of 95% ethanol. Aliquots of 800 μl of the resulting mixture from the tube was transferred to a column in a collection tube and centrifuged at 10,000 x g for 1 minute. To this, 400 μl ZymoBIOMICS DNA Wash Buffer 1 was added in the Zymo-Spin IICR Column in a new Collection Tube and centrifuged at 10,000 x *g* for 1 minute: removing the flow-through. A further 700 μl ZymoBIOMICS DNA Wash Buffer 2 was added to the Zymo-Spin IICR Column in a collection tube and centrifuged at 10,000 x *g* for 1 minute: this time discarding the flow-through. Following this, a 200 μl aliquot of ZymoBIOMICS DNA Wash Buffer 2 was added to the Zymo-Spin IICR Column in a collection tube and centrifuged at 10,000 x *g* for 1 minute with final discarding of the flow-through. The wash step with the ZymoBIOMICS DNA Wash Buffer 2 was repeated one more time. Finally, we transferred the Zymo-Spin IICR Column to a clean 1.5 ml PCR tube and add 100 μl Molecular Biology Grade Water, Corning^TM^ directly to the column matrix and incubated the setup for 5 minutes at room temperature. Final DNA extracts were eluted by spinning at 10,000 x *g* for 1 minute.


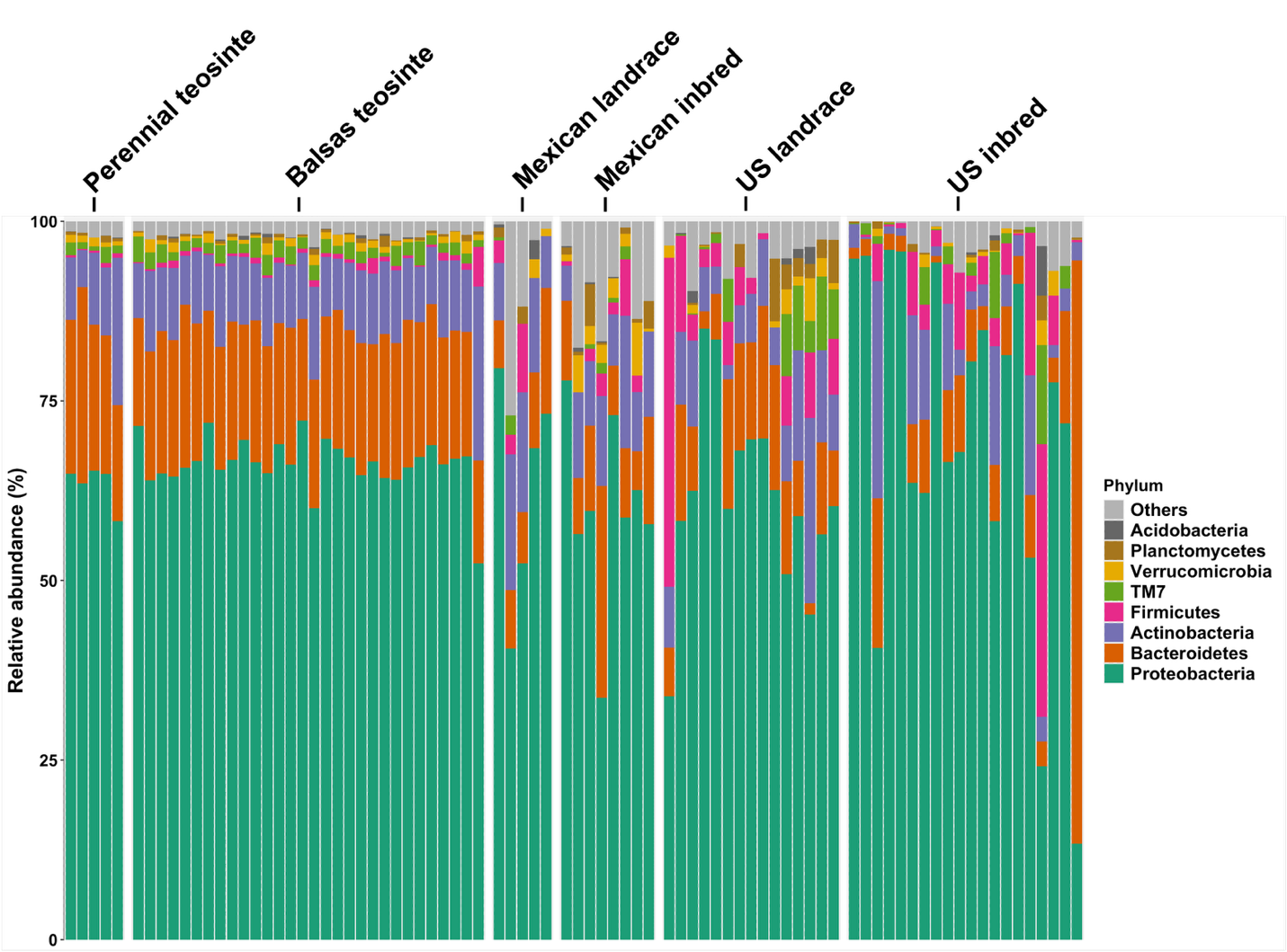


Fig. S1 Stacked bar plot illustrating taxonomic relative abundance (%) of leaf endosphere community composition at the phylum level. Only the most abundant taxa are shown. Each vertical bar represents an individual sample. The samples are categorized based on the maize genotypes indicated at the top. Proteobacteria (65.5%), Bacteroidetes (14.1%), Actinobacteria (9.26%), Firmicutes (3.4%) and TM7 (1.92%) were the most dominant phyla in maize leaf endosphere bacterial communities.


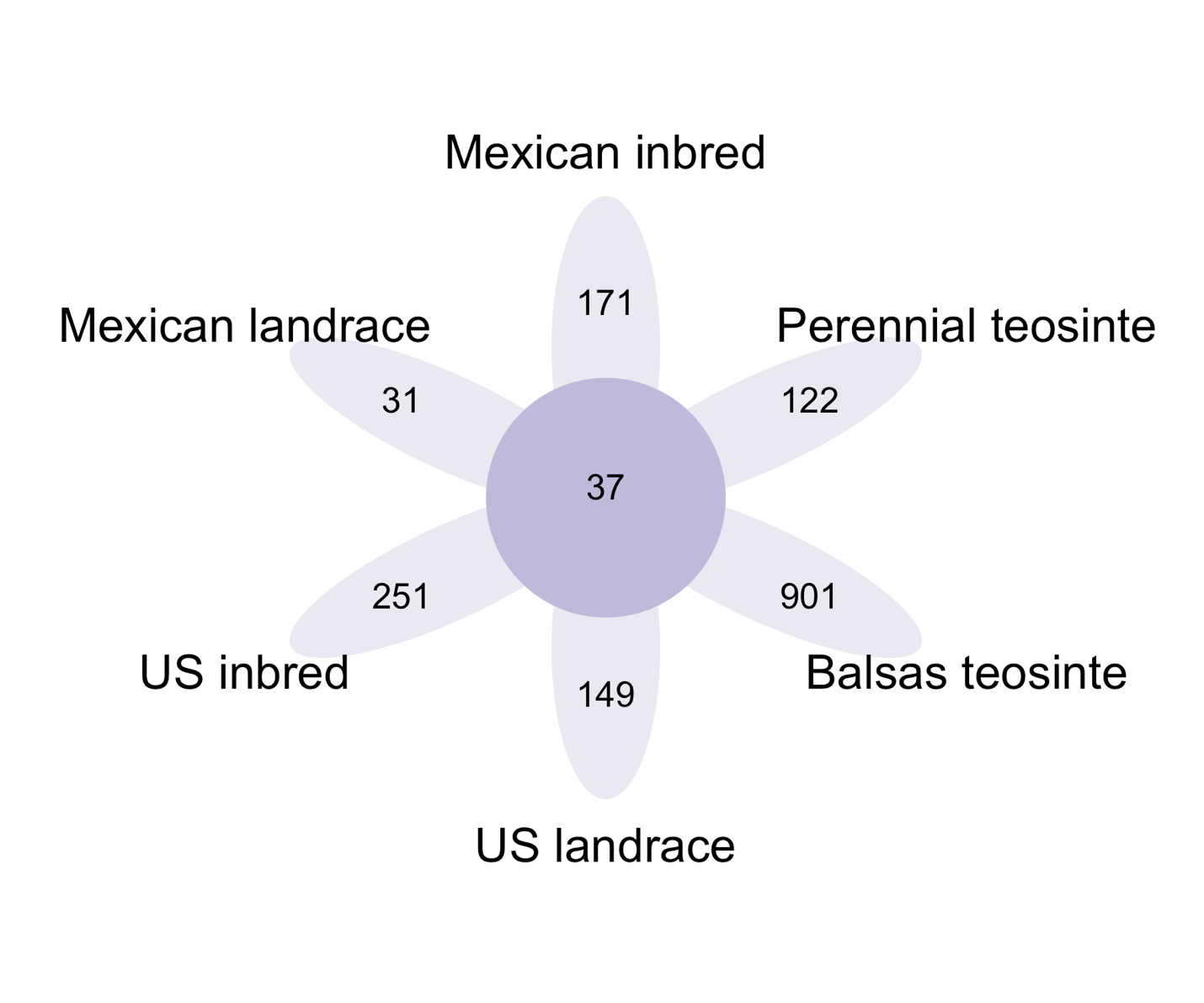


Fig. S2 Venn diagram showing the number of shared and unique OTUs between maize genotypes in the leaf endosphere microbiota. The core microbial community of the genotypes consisted of 37 OTUs. Perennial teosinte harbored 122 unique bacterial taxa, Balsas teosinte 901, US inbred 251, US landrace 149, Mexican inbred 171, and Mexican landrace 31, respectively.


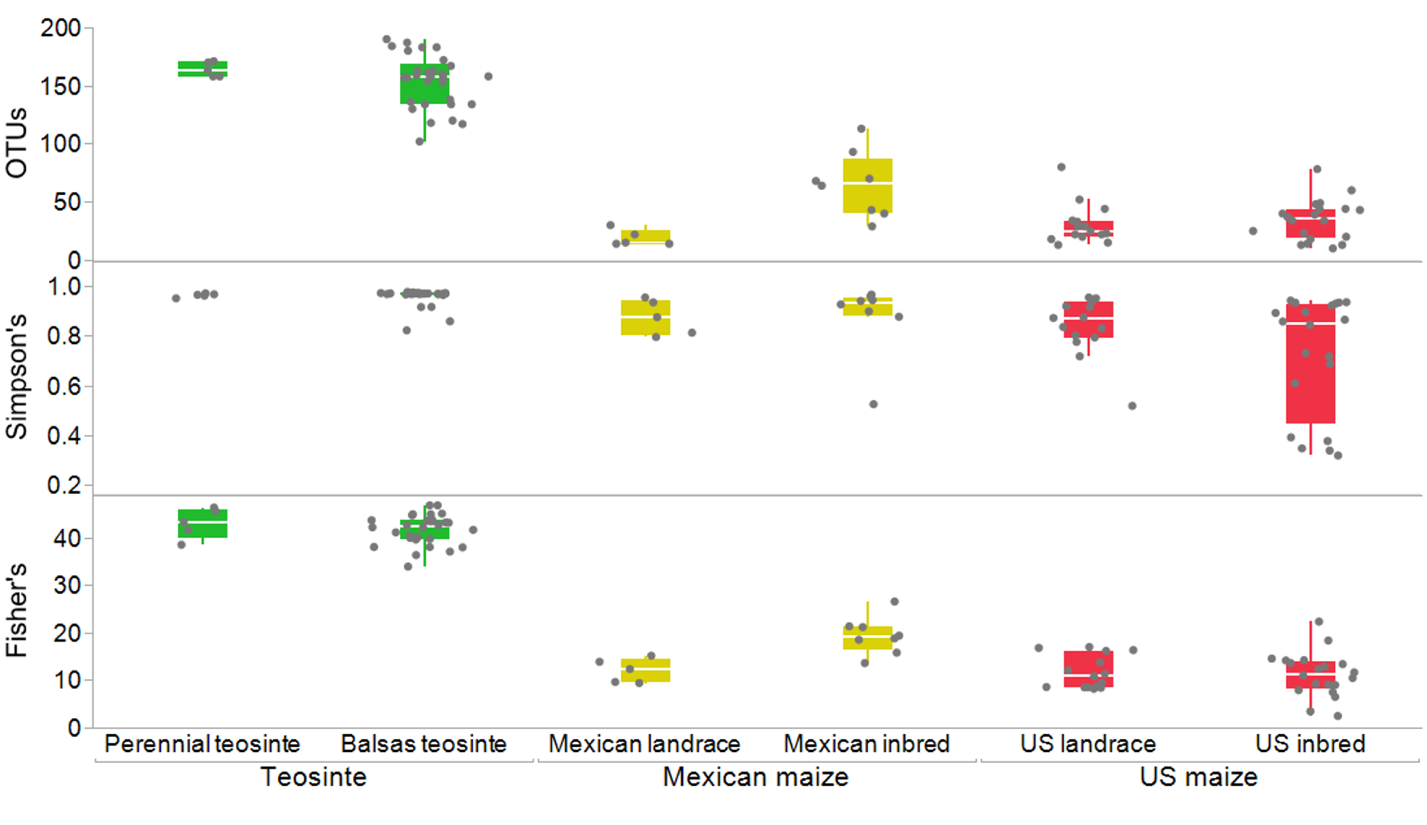


Fig. S3 Alpha diversity indices of the bacteria in leaf endosphere among maize genotypes. Box plots showing the number of richness OTUs, Simpson and Fisher diversity indexes, respectively. Comparisons of richness OTUs and Simpson and Fisher diversity indexes of leaf endosphere microbiota across the groups revealed a trend of decreasing diversity from teosinte to Mexican maize (landrace and elite inbred) and US maize (landrace and elite inbred). There was also a consistent decline in diversity from Balsas teosinte to Mexican landrace, suggesting the influence of domestication.


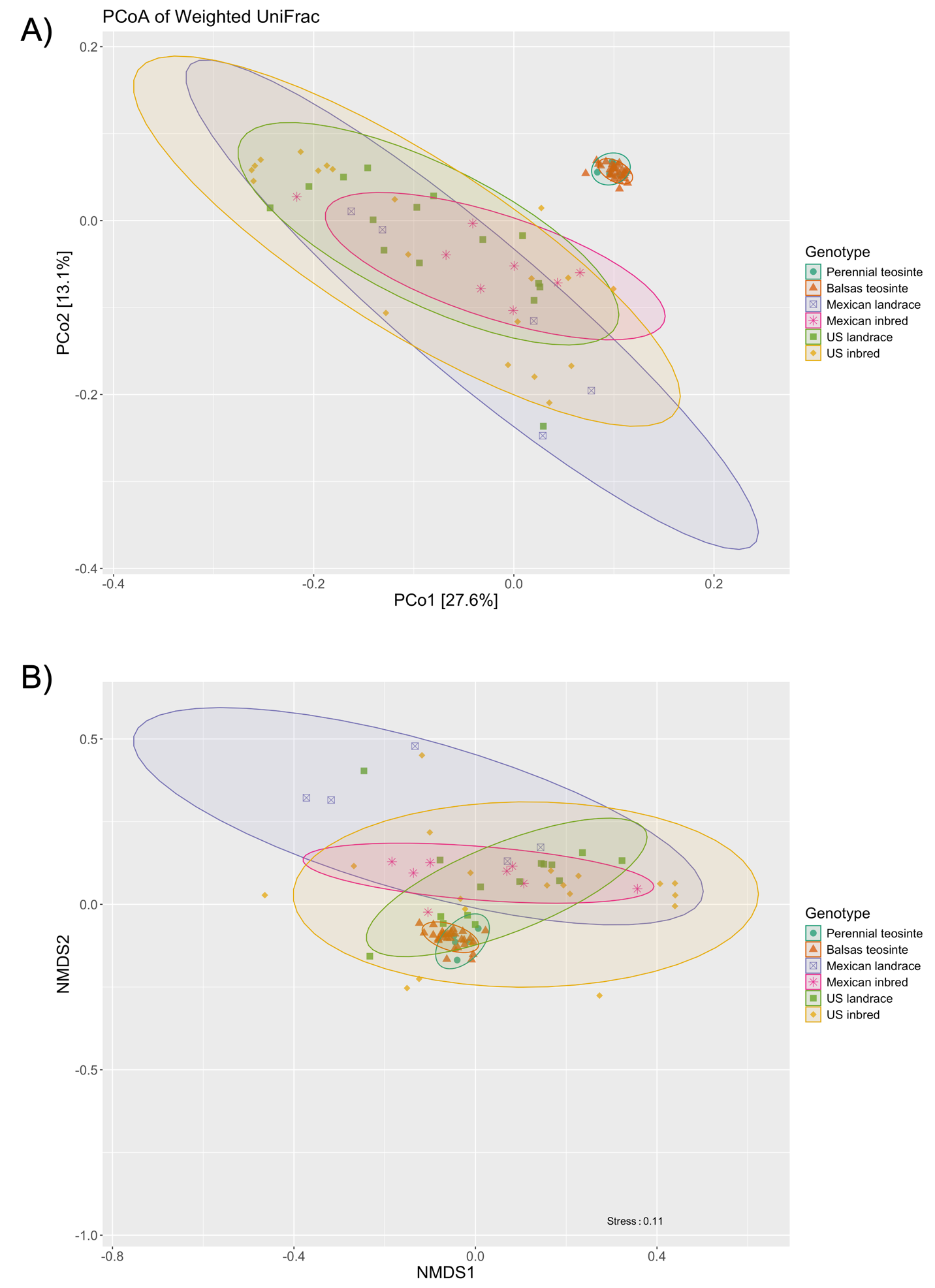


Fig. S4 Principal coordinates analysis (PCoA) of Weighted UniFrac (A) and non-parametric multivariate analysis of variance (NMDS) (B) of maize leaf endosphere microbiota. Each point represents a single sample, and colors indicate individual maize genotype. The Weighted Unifrac and NMDS analysis graphs clearly show a distinct divergence between teosinte and maize in variation.

#
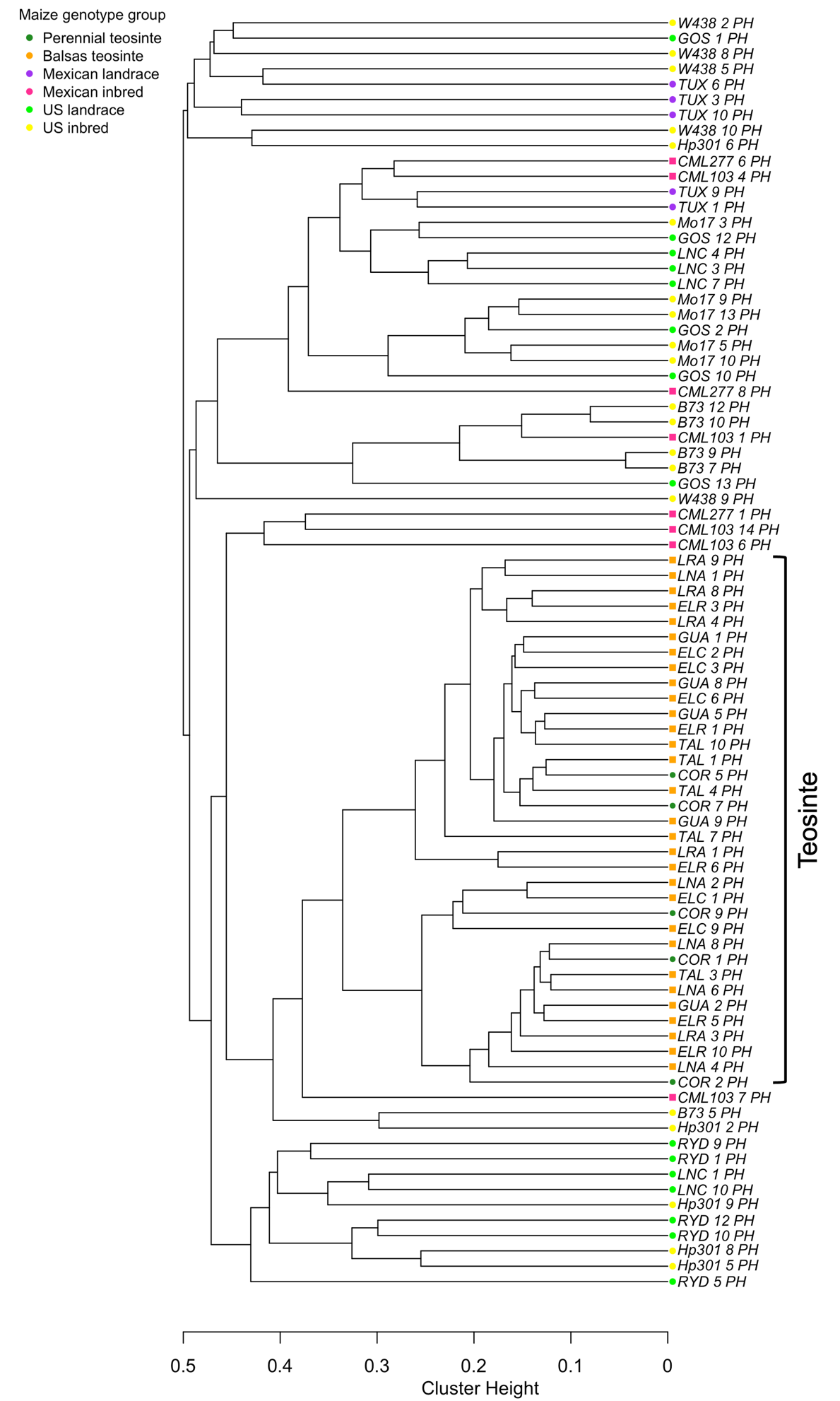


# Fig. S5 Cluster-analysis dendrogram of Bray-Curtis distances based on OTU abundances profiles in maize leaf endosphere across all the tested maize genotypes. The dotted line across the dendrogram shows a distinct separation between maize samples.

**
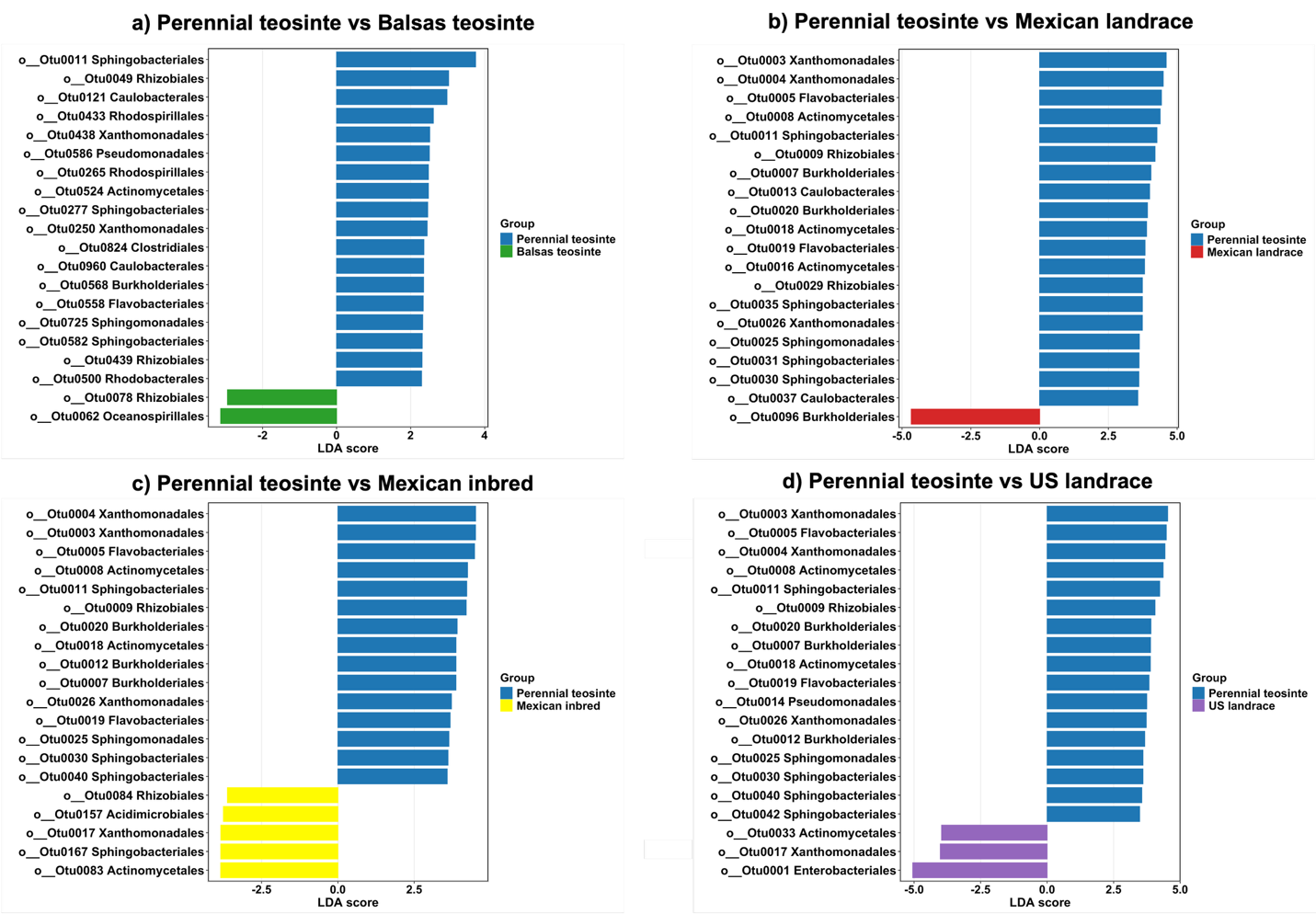
**


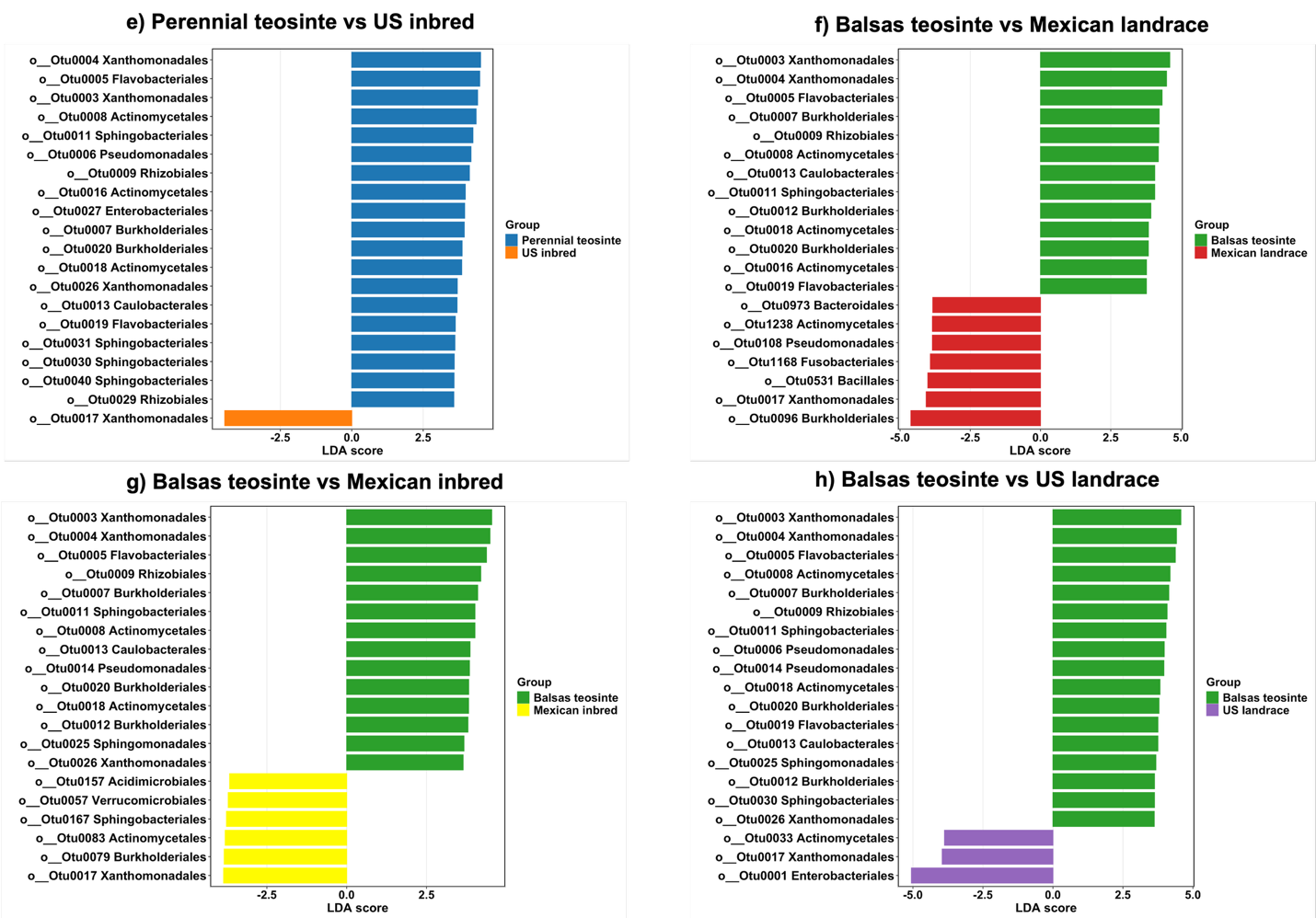


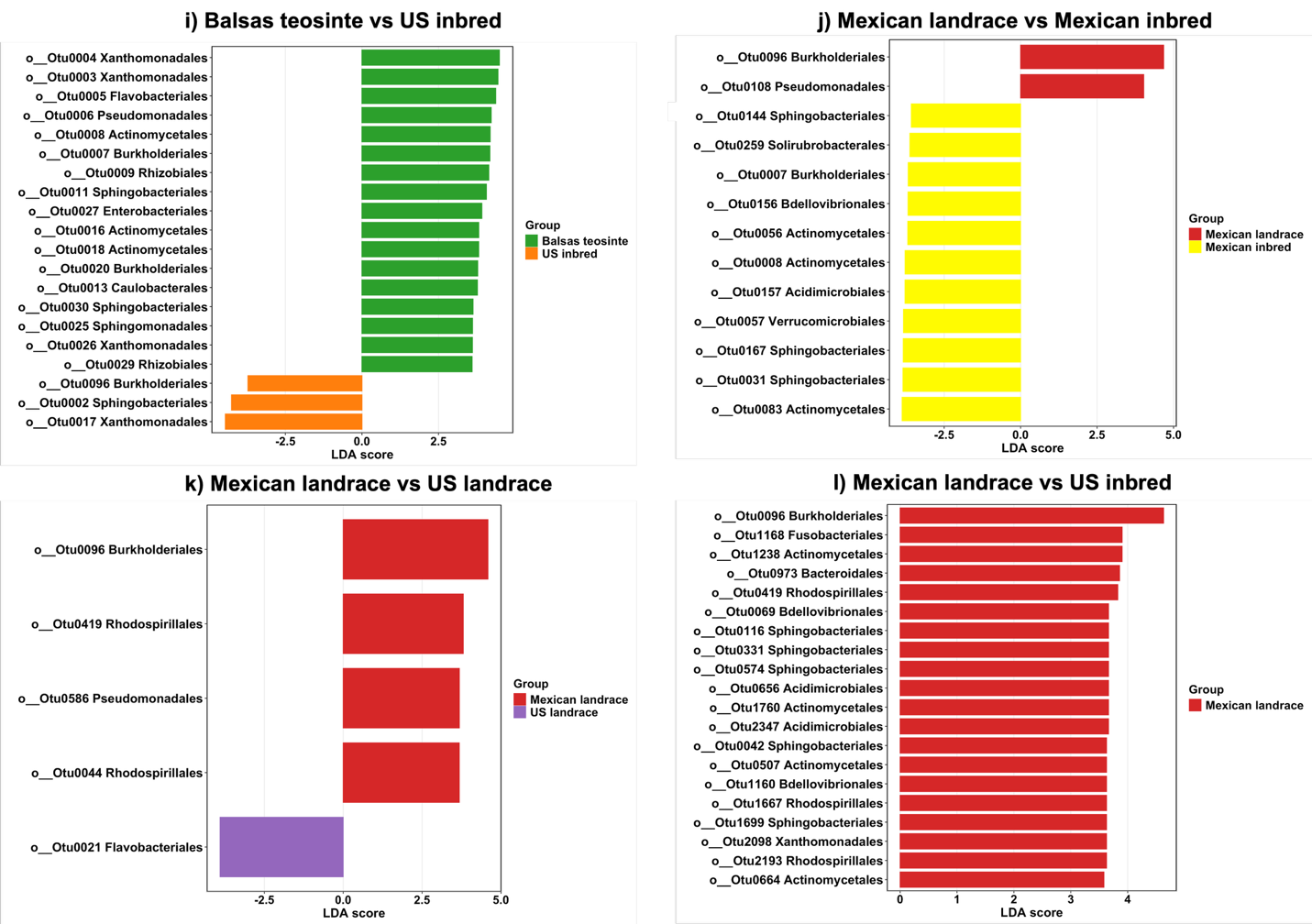


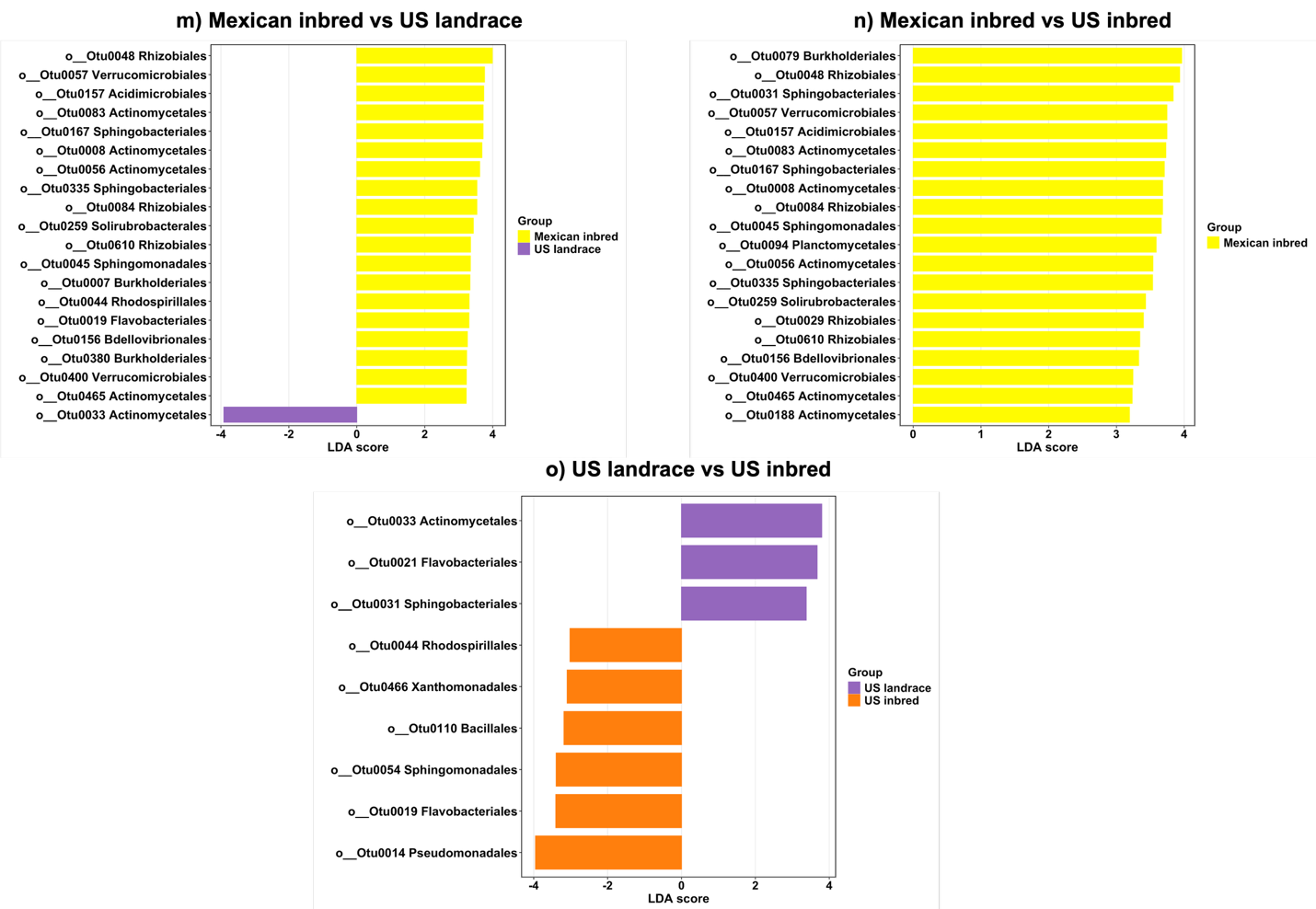


Fig S6 Linear discriminant analysis (LDA) effect size (LEfSe) analysis of microbial biomarker taxa in maize leaf endosphere microbiota among maize genotypes. Comparative analyses of taxonomic biomarkers were conducted among genotypes. .  Taxon's LDA score and enriched taxa are represented by horizontal bars, while colors indicate maize genotype. Comparisons based on the Kruskal-Wallis rank-sum test (α = 0.05), and taxa were ranked according to their LDA (log_10_) effect sizes. The LEfSe analysis for each genotype comparison revealed that most of the differentially abundant taxa were enriched in the teosinte genotypes, including both perennial and Balsas teosinte.
